# Delineation of an insula-BNST circuit engaged by struggling behavior that regulates avoidance in mice

**DOI:** 10.1038/s41467-021-23674-z

**Published:** 2021-06-11

**Authors:** Joseph R. Luchsinger, Tracy L. Fetterly, Kellie M. Williford, Gregory J. Salimando, Marie A. Doyle, Jose Maldonado, Richard B. Simerly, Danny G. Winder, Samuel W. Centanni

**Affiliations:** 1 Vanderbilt Center for Addiction Research, Vanderbilt University School of Medicine, Nashville, TN USA; 2 Vanderbilt Brain Institute, Vanderbilt University School of Medicine, Nashville, TN USA; 3 Vanderbilt J.F. Kennedy Center for Research on Human Development, Vanderbilt University School of Medicine, Nashville, TN USA; 4 Department of Molecular Physiology & Biophysics, Vanderbilt University School of Medicine, Nashville, TN USA; 5 Department of Psychiatry and Behavioral Sciences, Vanderbilt University School of Medicine, Nashville, TN USA

**Keywords:** Neuroscience, Neural circuits, Stress and resilience

## Abstract

Active responses to stressors involve motor planning, execution, and feedback. Here we identify an insular cortex to BNST (insula^→BNST^) circuit recruited during restraint stress-induced active struggling that modulates affective behavior. We demonstrate that activity in this circuit tightly follows struggling behavioral events and that the size of the fluorescent sensor transient reports the duration of the struggle event, an effect that fades with repeated exposure to the homotypic stressor. Struggle events are associated with enhanced glutamatergic- and decreased GABAergic signaling in the insular cortex, indicating the involvement of a larger circuit. We delineate the afferent network for this pathway, identifying substantial input from motor- and premotor cortex, somatosensory cortex, and the amygdala. To begin to dissect these incoming signals, we examine the motor cortex input, and show that the cells projecting from motor regions to insular cortex are engaged shortly before struggle event onset. This study thus demonstrates a role for the insula^→BNST^ pathway in monitoring struggling activity and regulating affective behavior.

## Abstract (web summary)

Active responses to stressors involve motor planning, execution, and feedback. The authors identify a neuronal projection from the insular cortex to the bed nucleus of the stria terminalis that is activated during motor struggling in response to restraint stress as a potential active coping response.

## Introduction

The response to stress is critical to every organism’s capacity to adapt and survive. An appropriate stress response shifts metabolic resources toward critical systems involved in the threat perception and the subsequent fight-or-flight response. Active stress coping relies on an individual’s resources to escape a stressor, while passive stress coping relies on external factors to end the stressor^1^. In humans, active coping is thought to be a crucial component of resilience^1^. Evidence also exists in animals that active coping can change the behavioral and physiological response to a stressor^2^. The antecedent effects of stress on neuronal activity in many brain regions and the real-time effects of stress in the paraventricular nucleus have been examined^3–5^; however, the circuits that participate in the real-time reporting of selected active stress-coping strategies have not been determined.

Integration of active stress-coping activity into a maintained stress response necessarily entails coordination of interoceptive and stress-axis circuitry. The insula is an ideal candidate, given its role in both interoception and affect^6^. Moreover, the insula shares dense interconnections with stress-related regions, such as the bed nucleus of the stria terminalis (BST, BNST), which plays a major role in modulating the hypothalamic–pituitary–adrenal (HPA) axis and has been implicated in passive stress-coping behavior^7^. In addition, recent studies have indicated that optogenetic stimulation of the posterior insula produces avoidance-related behaviors^8^, and given the BNST’s role in avoidance behavior^9^, an insula–BNST circuit could also play an integral role in active escape behavior during a stressor.

In many stress models, animals actively cope through physical motor activity. Physical activity can regulate multiple aspects of central nervous system (CNS) function^10^. Despite posited relationships between physical activity and affective state, functional paths between executive motor regions and forebrain structures encoding this relationship have not been clearly defined. Moreover, how, or if, these functional motor pathways mediate stress responses and coordinate affective state remains unclear.

In this study, we elucidated a mid-insula:BNST^CRF^ neuronal circuit (insula^→BNST^) that reports struggling behavior during restraint stress, a potential active coping response, and show this circuit regulates subsequent affective behavior. Further, we demonstrate that the size of the calcium transient in this pathway correlates with the duration of physical struggle, and that this correlation degrades with habituation to a homotypic stressor. While the fidelity between insula^→BNST^ calcium transient and physical struggles decayed during stress habituation, insular glutamate transient-physical activity fidelity did not, suggesting that habituation may occur downstream of insular afferents. We performed unbiased retrograde and anterograde whole-brain mapping of neuronal connections to determine the afferent network for insula^→BNST^ neurons. In doing so, we uncovered an unexpectedly large input from executive motor cortical regions and demonstrated that activity in these insula-projecting motor cortex neurons is time-locked to and precedes the onset of the restraint stress struggle events and insula^→BNST^ activity.

## Results

### BNST neurons are activated at the onset of struggling bouts during restraint stress

**Fig. 1:**
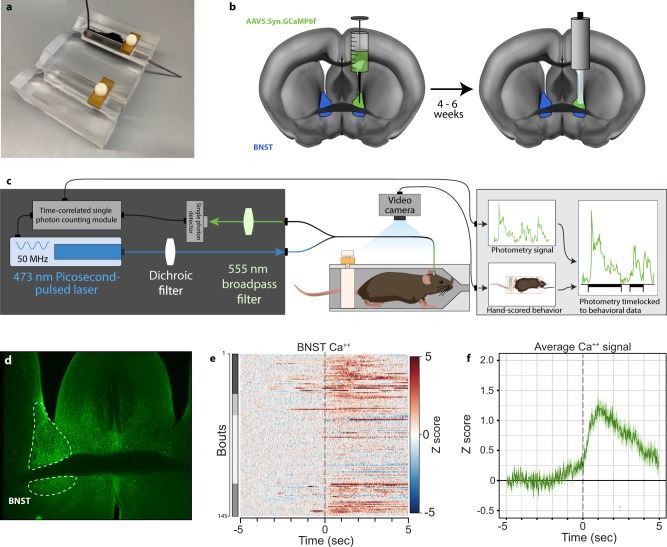
Struggling bouts during restraint stress are associated with calcium transients in the BNST. **a** Image of RESTRAINT (*RE*cording *S*ignal *TR*ansients *A*ccessible *IN* a *T*ube) device for simultaneous fiber photometry and behavior recordings. **b** Diagram of AAV5.hSyn.GCaMP6f injection and fiberoptic implant into the BNST. Animals were tested 4–6 weeks after fiber implantation. Schematic of fiber photometry with RESTRAINT device and behavioral scoring pipeline. **c**, **d** Representative image of GCaMP expression in the BNST. **e** Heatmap of bout-associated GCaMP transients in the BNST. For all heatmaps, photometry signals run horizontally. Signals were time-locked to bout onset and were aligned such that bout onset began at 0 s on the *x*-axis. Red indicates an intensity increase, and blue indicates a decrease relative to baseline. Values beyond the max/min values noted on the color legend are displayed with the color at the legend’s respective ends. The total number of traces for each heatmap is displayed at the graph’s far left (i.e., 1 through the total trace number). Data from individual animals are grouped in this plot, as indicated by distinct gray shaded bars along the left axis (*n* = 4 mice, 145 total bouts). **f** Average time-locked GCaMP signal from all bouts. Lighter shading: s.e.m. across all bouts.

**Fig. 2:**
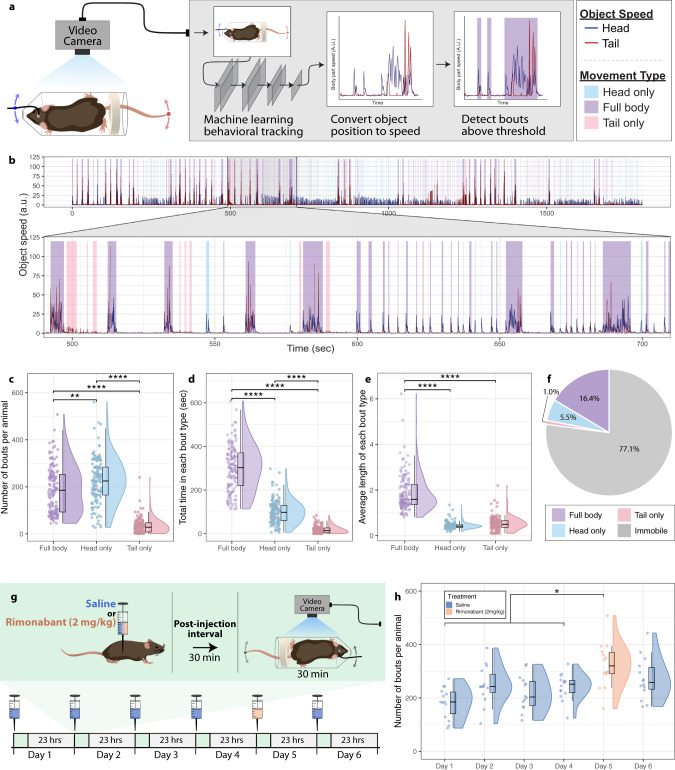
Computer vision can capture struggling bouts during restraint stress and show that bout number increases with rimonabant. **a** Pipeline schematic. Head-attached fiber (blue dot) and the tail tip (red dot) were tracked. **b** Example output from 30-min restraint test. Head movement speed (dark blue) and tail movement speed (dark red). Shading colors: full body (purple), head (light blue), and tail (light red). **c**–**f** Data from 136 trials (30-min each) with a total of 60,456 bouts. Each point represents the average number of bouts from one 30-min trial. Purple: full body movements. Light blue: head only movements. Light red: tail only movements. **c** Movement bouts by type. One-way ANOVA *F*_(2, 397)_ = 200.3, *P* < 0.0001; Bonferroni post hoc analysis: **P = 0.0014 for full body vs. head only; ****P < 0.0001 for full body vs. tail only; ****P < 0.0001 for head only vs. tail only. **d** Total time spent in each bout type. One-way ANOVA *F*_(2, 397)_ = 587.2, *P* < 0.0001; Bonferroni post hoc analysis: ****P < 0.0001 for full body vs. head only; ****P < 0.0001 for full body vs. tail only; ****P < 0.0001 for head only vs. tail only. **e** Length of each bout type. One-way ANOVA *F*_(2, 397)_ = 335.6, *P* < 0.0001; Bonferroni post hoc analysis: ****P < 0.0001 for full body vs. head only; ****P < 0.0001 for full body vs. tail only; *P* = 0.47 for head only vs. tail only. **f** Percent of trial spent on each bout type. Light gray: immobile. **g** Schematic. **h** Mean number of detected full body, overlapping head and tail movement bouts, (purple bars from **b**) by day. (*n* = 14 mice). Mixed-effects model Dunnett’s multiple comparisons test ***P = 0.0002 for day 1 vs. day 5; *P = 0.0288 for day 2 vs. day 5; **P = 0.0025 for day 3 vs. day 5; **P = 0.0048 for day 4 vs. day 5; *P* = 0.1138 for day 6 vs. day 5. Colors: i.p. saline (blue) or rimonabant (peach). **c**–**e**, **h** Distribution indicated with split violin normalized to width. Median, quartiles, and 1.5 × the interquartile range are indicated with the associated box plot.

We investigated BNST neuronal activity during acute restraint stress. Restraint stress is a widely used stressor in rodents and a pharmacologically validated model for inducing negative affect^11^ and engaging BNST signaling^12^. During restraint stress, rodents engage in periodic physical struggle events (i.e., escape behavior) that are thought to underlie active stress coping^11,13^. Compounds known to increase anxiogenic behavior in rodents, such as the anxiogenic cannabinoid 1 (CB_1_) receptor antagonist rimonabant, significantly increase this struggling behavior and also elevate the expression of the immediate-early gene *fos* in the BNST^11^, suggesting a link between struggling behavior and increased BNST activity. We engineered a custom restraint stress apparatus (RESTRAINT, *RE*cording *S*ignal *TR*ansients *A*ccessible *IN* a *T*ube) to enable concurrent behavior and fiber photometry recordings (Fig. 1a and Supplementary Fig. 1a–e). This simultaneous recording permitted the direct comparison of time-locked active struggling behavior to calcium transients measured in the BNST (Fig. 1b–d). Consistent with previous *fos* data, BNST calcium transients tightly aligned with struggling bout onset (Fig. 1e, f), while offsetting the behavioral alignment by +10 s eliminated this association (Supplementary Fig. 1f, g). Next, to increase the throughput and reproducibility of struggling bout analysis, we implemented computer vision-based behavioral scoring using DeepLabCut (DLC)^14,15^ (Fig. 2a, b). We recorded behavior during restraint stress from 136 trials of 36 mice used in this study, analyzed 60,456 total movement bouts, and categorized struggle types into either head only, tail only, or a combination of head and tail movements (full body movements). Head only movements were more frequent than tail only and full body (Fig. 2c). However, the cumulative time spent engaging in these behaviors, and the average length (duration) of the bouts were highest for the full body struggles (Fig. 2d, e). Thus, analyses here focused on full body struggles as these produced the most robust behavioral response, requiring coordinated head and tail movement. Of note, all struggling behavior is rare relative to the duration of the entire restraint stress assay (Fig. 2f), suggesting this behavior marks unique timepoints during restraint stress, where mice initiate and sustain stress-responsive behavior. To determine if DLC-based scoring could accurately detect changes in anxiogenic behavior, we subjected mice to 30 min of restraint stress on six consecutive days. On each of the first four days, we injected mice with saline prior to restraint stress. On the fifth day, we injected the mice with the CB_1_ receptor antagonist, rimonabant^11,16,17^ (i.p. 2 mg/kg, a dose that does not alter locomotor activity^18^; Fig. 2g). Rimonabant increased the number of algorithm-detected struggling bouts compared to previous saline injection days. Moreover, when saline was administered prior to restraint stress on day six, struggling bouts were no longer elevated, indicating that our DLC-based system could accurately measure salient anxiogenic behaviors during restraint stress (Fig. 2h).

**Fig. 3:**
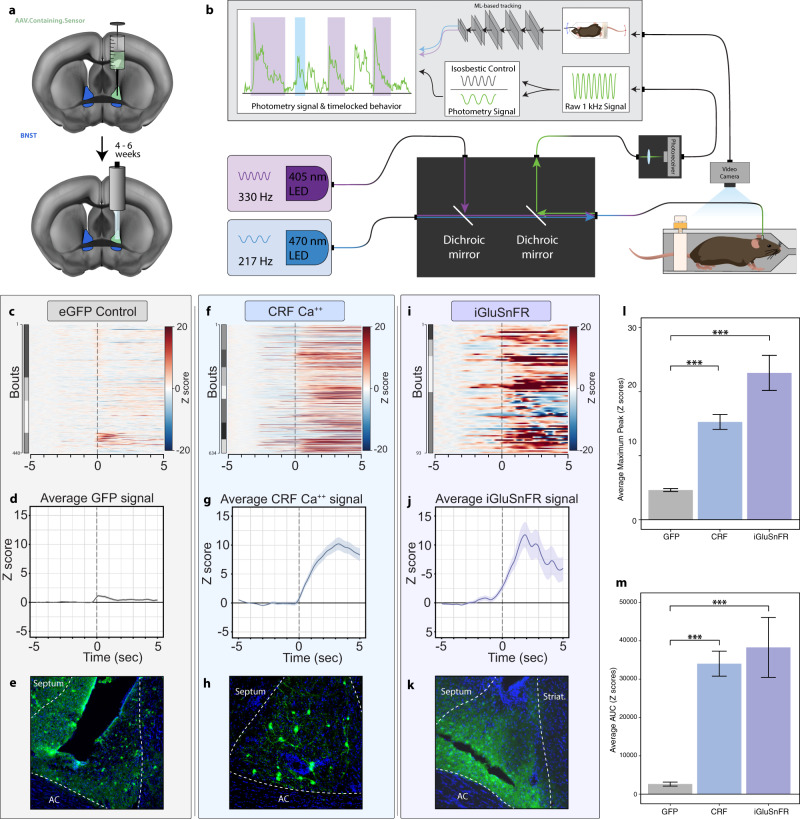
Calcium signals from BNST^CRF^ neurons and BNST glutamate transients are associated with struggling bouts. **a** Diagram of sensor-containing virus injection and fiber photometry implant into the BNST. Viral constructs coded for either eGFP control, Cre-dependent GCaMP, or SF-iGluSnFR. **b** Schematic of two-channel fiber photometry system with the ability to implement an isosbestic control. Signal is time-locked to machine learning-based scoring of RESTRAINT device behavior. **c**–**e** Photometry recording from mice that received an injection of AAV5.eGFP in the BNST. **c** eGFP control heatmap of time-locked bouts. **d** Average eGFP signal. Lighter shading: s.e.m. across all bouts. **e** Example image of eGFP expression in the BNST. **f**–**h** Photometry recording from CRF-Cre mice that received an injection of Cre-dependent GCaMP in the BNST. **f** Heatmap of Cre-dependent GCaMP signal in CRF-Cre mice that was time-locked to bouts. **g** Average Cre-dependent GCaMP signal in CRF-Cre mice. Lighter shading: s.e.m. across all bouts. **h** Example image of Cre-dependent GCaMP expression in CRF-Cre mice. **i**–**k** Photometry recording from mice that received an injection of AAV1.SF-iGluSnfR in the BNST. **i** Heatmap of SF-iGluSnFR signal time-locked to bouts. **j** Average SF-iGluSnFR signal. Lighter shading: s.e.m. across all bouts. **k** Example image of SF-iGluSnFR expression in the BNST. **l** Average maximum peak *Z* score of CRF-GCaMP (*n* = 634 bouts from nine mice) and SF-iGluSnFR (*n* = 93 bouts from four mice) were higher than eGFP (*n* = 440 bouts from four mice) controls. One-way ANOVA *F*_(3,1744)_ = 40.23, *P* < 0.0001; Bonferroni post hoc analysis: ***P < 0.0001 for eGFP vs. CRF-GCaMP; ***P < 0.0001 for eGFP vs. SF-iGluSnFR. Error bars: s.e.m. across bouts. **m** Average AUC *Z* score of CRF-GCaMP and SF-iGluSnFR were higher than eGFP controls (*n* = same as **l**). One-way ANOVA *F*_(3,1744)_ = 31.98, *P* < 0.0001; Bonferroni post hoc analysis: ***P < 0.0001 for eGFP vs. CRF-GCaMP; ***P < 0.0001 for eGFP vs. SF-iGluSnFR. Error bars: s.e.m. across bouts.

The BNST is composed of many subtypes of neurons, including a dense population of cells expressing the neuropeptide corticotropin-releasing factor (CRF). BNST^CRF^ neurons have been identified as stress-responsive cells that drive affective behaviors^19–21^, and acute stress models, such as restraint stress reliably induce increases in *fos* activity in CRF cells that persist after the stress has ended^22,23^. BNST^CRF^ activity during a stressor has not been assessed to date. To explore signaling specifically within BNST^CRF^ cells, we unilaterally injected the BNST of CRF-Cre mice with a Cre-dependent GCaMP7f adeno-associated virus (AAV). We then simultaneously recorded behavior and in vivo calcium signaling during restraint stress (Fig. 3a, b). Struggling bouts were associated with increased calcium transients in BNST^CRF^ cells (Fig. 3f–h). Struggling bout duration was positively correlated with calcium transient area under the curve (AUC) and maximum peak amplitude (Supplementary Fig. 2p, q). Further, there were significantly more struggle bouts with positive calcium transients (**P = 0.0025 for AUC) than there were without (Supplementary Fig. 2r, s), suggesting that while BNST^CRF^ calcium transients are not exclusive to struggle bouts, as a whole, the transients are positively correlated with the duration of the bouts. To assess the specificity of the BNST^CRF^ response, we compared it to signals associated with struggling behavior from a distinct population of BNST^PKCδ^ cells (Supplementary Fig. 2a–e), and found that the BNST^CRF^ response was significantly larger (Supplementary Fig. 2f, g). Because stress can increase glutamatergic signaling in the BNST^24^, we incorporated a direct measurement of extracellular glutamate by utilizing the fluorescence intensity-based glutamate sensor, SF-iGluSnFR^25^. This extracellular glutamate sensor revealed an increase in extracellular glutamate at BNST neurons at the onset of struggling bouts (Fig. 3i–k).

Because struggling bouts are movement dependent, the observed signals could involve movement artifacts. Therefore, we tested this possibility with a number of experiments. (1) As an internal control, we used a photometry system that measures calcium-independent isosbestic GCaMP signal, allowing for the subtraction of movement artifacts from the calcium-dependent GCaMP signal (Fig. 3b). (2) We injected a stable, activity-independent fluorophore (eGFP) in lieu of a sensor (Fig. 3a–e). Minimal time-locked signals were seen across eGFP animals, suggesting movement had only a very small contribution to the overall signal we observed. BNST^CRF^ and SF-iGluSnFR sensors generated much higher average peak transient amplitudes and AUC from 0–5 s compared to the eGFP control (Fig. 3l, m). (3) We also identified calcium signaling in the insula that was negatively correlated with movement. We trained mice expressing GCaMP in the insula to run on a running wheel, while simultaneous photometry signals were measured. While the initiation of running induced a time-locked increase in calcium transient amplitude, transient frequency was significantly lower overall when the mice were running than when they were not (Supplementary Fig. 3). (4) We reliably observed struggle bouts that failed to initiate an increase in sensor photometry signal (nonpositive AUC after bout onset, see below), collectively negating the possibility of fiber movement in the brain as a driver of the signal.

To test for uncorrelated activity, we offset the behavior-photometry time lock by +10 s for all eGFP, GCaMP, and SF-iGluSnFR experiments. In each case, transient signal decreased or was eliminated altogether (Supplementary Fig. 2h–o). In total, these findings demonstrate that BNST^CRF^ neurons are recruited in tight association with struggling events, and that increased glutamatergic drive onto BNST cells could be responsible for the changes in calcium transients that we observed in select stress-sensitive neuronal populations.

### The insula is engaged by acute restraint stress and modulates stress-related BNST activity

Given the large glutamatergic transients observed in the BNST associated with struggling bouts, coupled with our previous work indicating that insular afferents release glutamate at synapses on BNST^CRF^ neurons^22,26^, we explored the influence of this projection on BNST activity during stress. We first used retrograde and anterograde viral tracing strategies in combination with light sheet-based whole-brain mapping to better characterize the distribution of these BNST afferents (Supplementary Fig. 4a–c). We identified the middle insula as the densest insular input to the mouse BNST (Supplementary Fig. 4d–i and Supplementary Movies 1–4)^27^, and the oval, juxtacapsular, rhomboid, and anterolateral subnuclei of the BNST (Supplementary Fig. 4l–o) as major sites of insular afferent targeting in the region.

**Fig. 4:**
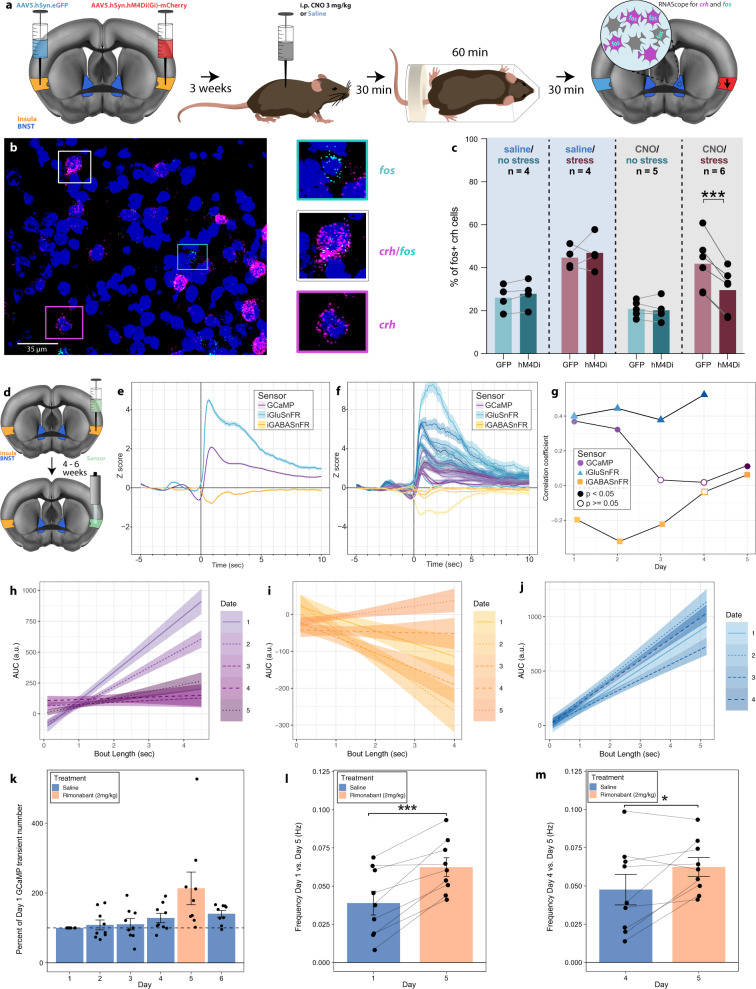
Insular G_i_-DREADD activation decreases BNST *fos* expression after restraint stress, and insular transients are associated with struggling bouts. **a** Timeline. Mice were injected with a G_i_-DREADD (hM4Di) coding virus in one hemisphere (red) and an eGFP control in the other (cyan). Three weeks later, mice were injected with CNO (3 mg/kg i.p.). After 30 min, they were restrained for 1 h. Animals were processed 30 min later for fluorescent in situ hybridization. **b** Example fluorescent in situ hybridization from the BNST with examples of *crh* only (magenta box), *fos* only (cyan box), and *crh/fos* doubly labeled (white box) cells. **c** Comparison of restraint stress (burgundy) vs. nonstress (teal) and/or CNO (gray) or saline (blue) exposure on percent of *fos*-positive *crh* cells in the BNST ipsilateral to the G_i_-DREADD injection (darker shading) vs. the BNST ipsilateral to the eGFP control (lighter shading). Paired *t* test (two-sided), *t*_(5)_ = 7.122, ***P = 0.0008. **d** Schematic. Virus coding for either GCaMP7f (*n* = 9), SF-iGluSnFR (*n* = 10), or iGABASnFR (*n* = 5) was injected into the insula, followed by fiber placement. **e** Bout-associated signals from GCaMP (purple), SF-iGluSnFR (blue), or iGABASnFR (yellow) across six 30-min restraint exposures (see Fig. 2g). Lighter shading: s.e.m. across bouts. **f** Bout-associated signals by animal. **g** Correlation coefficient (Pearson, no adjustments for multiple comparisons) of the relationship between AUC from 0 to 5 s and bout length over days. GCaMP (purple circle), SF-iGluSnFR (blue triangle), and iGABASnFR (yellow square). Correlation coefficient shape is solid if *P* < 0.05 and open if *P* ≥ 0.05. Rimonabant was administered to GCaMP and iGABASnFR on day 5. SF-iGluSnFR experiment finished on day 4. **h**–**j** Slope of AUC vs. bout length by day with 95% confidence interval. **h** GCaMP (purple). **i** SF-iGluSnFR (blue). **j** iGABASnFR (yellow). **k** Percent of GCaMP transient number normalized to day 1 (set at 100%). (*n* = 9) Error bars: s.e.m. across animals. Colors: saline (blue) and rimonabant (peach). **l** Frequency of GCaMP transients during restraint on day 1 (30 min after i.p. saline injection) vs. day 5 (30 min after i.p. rimonabant injection). Paired *t* test (two-sided), *t*_(8)_ = −5.4881, ***P = 0.0006. **m** Frequency of GCaMP transients during restraint on day 4 (30 min after i.p. saline injection) vs. day 5 (30 min after i.p. rimonabant injection). Paired *t* test (two-sided), *t*_(8)_ = −2.3889, *P = 0.043. **l**, **m** Error bars: s.e.m. across bouts (*n* = 9).

To test the role of insular inputs in modulating BNST^CRF^ neuron stress responses, we injected the mid-insula of one hemisphere with a virus containing the G_i_-Designer Receptor Exclusively Activated by Designer Drug (DREADD) hM4Di, and an eGFP control virus contralaterally. We administered clozapine-*N*-oxide (CNO, i.p. 3 mg/kg) or saline 30 min prior to 60 min of restraint stress. We then quantified *fos* expression as a proxy for neuronal activity in BNST^CRF^ cells using fluorescent in situ hybridization (Fig. 4a, b). Consistent with our hypothesis, the percent of BNST^CRF^ cells that expressed *fos* after stress was significantly lower in the G_i_-DREADD-injected hemisphere relative to the GFP-injected hemisphere in CNO-treated mice (Fig. 4c).

Because of the effect of restraint on the BNST and the insula–BNST pathway, we next determined how restraint stress impacted the signaling within the insula by virally transducing GCaMP into the mid-insula (Fig. 4d). Average struggle bout-associated insular GCaMP signal increased at bout initiation. We used two fluorescent intensity-based neurotransmitter sensors, SF-iGluSnFR and iGABASnFR^28^, to examine the contribution of insular microcircuitry to struggle behavior. SF-iGluSnFR showed increased glutamate release onto insula neurons at the onset of a struggling bout. Conversely, iGABASnFR recordings demonstrated bout-associated signals deflected in the negative direction at bout onset (Fig. 4e, f), suggesting transiently decreased extracellular GABA. This pattern was consistent across mice (Fig. 4f). In addition, the duration of struggle bouts was positively correlated with AUC for insula GCaMP and SF-iGluSnFR signal, and negatively correlated with insula iGABASnFR (Fig. 4g, day 1 and Supplementary Fig. 6a, c, e). The maximum peak amplitude for GCaMP, SF-iGluSnFR, and iGABASnFR followed a similar pattern (Supplementary Fig. 7a, e, i). Further analysis revealed that a significantly higher number of bouts corresponded to increased calcium (*P = 0.0167 for GCaMP AUC) and glutamate signaling (****P < 0.0001 for SF-iGluSnFR AUC) than bouts that did not correspond with a change in signal. This suggests that while bouts with an associated increase in calcium/glutamate occur more frequently than bouts without an increase, insular calcium/glutamate changes are not uniformly dictated by struggling bouts (and vice versa), and are likely modulated by interdependent nodes of a larger related network. Insula neurons expressing iGABASnFR did not follow this pattern, as bouts were slightly more likely to occur with a transient AUC < 0 (*P* = 0.0607 for iGABASnFR AUC). The greater likelihood of bouts being associated with an iGABASnFR AUC < 0 is consistent with the negative deflection observed, when the signal is time-locked to the onset of struggle bouts.

### Homotypic stress habituation is associated with decreased fidelity of insula^→BNST^ struggle response

A hallmark of stress biology is that repeated homotypic stressor exposure leads to response habituation^29^. Thus, we examined the signals with each sensor across multiple stress days to test if there was a change in the relationship between photometry signal and behavior (Fig. 4g–j and Supplementary Figs. 6 and 7). We found that the GCaMP and iGABASnFR correlations between signal size and struggle duration collapsed across repeated days of exposure. Interestingly, however, insular SF-iGluSnFR signals remained elevated after repeated days of stress, suggesting habituation occurs postsynaptic to insular afferents (Fig. 4g, Supplementary Fig. 6e, f, and Supplementary Fig. 7i–l).

Given that rimonabant increased struggling bouts, we next tested whether the drug acts in part through increasing insular activity. SF-iGluSnFR signal did not habituate; therefore, we examined rimonabant’s effect on insular iGABASnFR and GCaMP signal. Rimonabant reestablished a positive correlation between AUC and bout length for GCaMP, but did not restore the correlation between iGABASnFR and bout length (Fig. 4g–i, and Supplementary Figs. 6 and 7). We then normalized insular calcium transient frequency over days 2–6 to each subject’s number of transients on day 1 (Fig. 4k). We compared GCaMP transient frequencies from the first restraint exposure (day 1) and the exposure immediately preceding rimonabant (day 4) to the frequencies observed, following rimonabant administration (day 5). Rimonabant significantly increased insular GCaMP transient frequency (Fig. 4l, m), suggesting the anxiogenic actions of rimonabant act in part through insular cortex signaling.

### Chemogenetically activating insula^→BNST^ neurons increases behaviorally time-locked BNST calcium signal during restraint stress and subsequent anxiety-like behavior

**Fig. 5:**
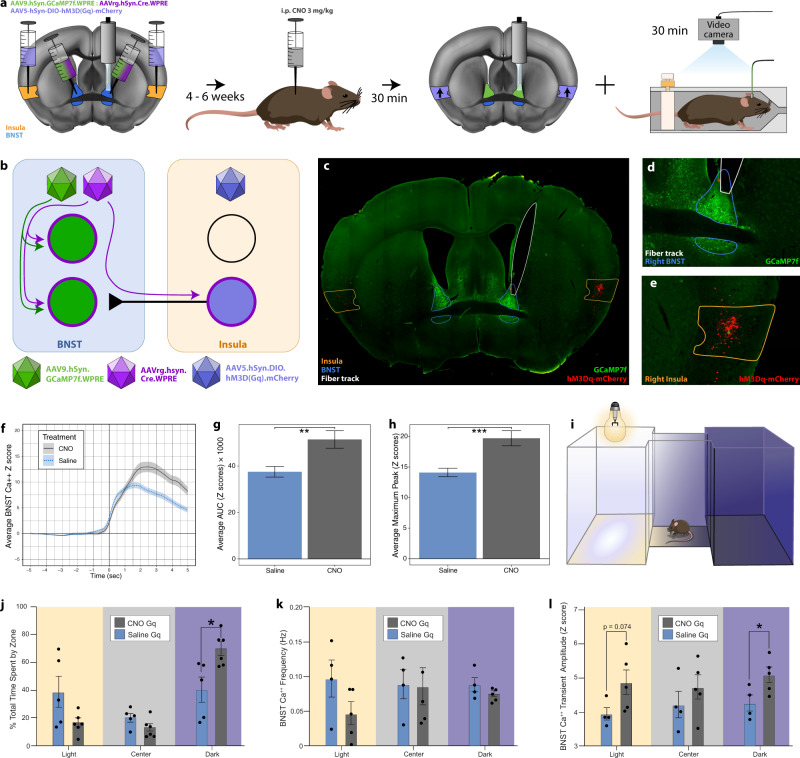
Chemogenetically activating insula^→BNST^ cells increases struggling bout-associated BNST calcium transients and anxiety-like behavior after restraint. **a**, **b** Schematics. **c** Representative coronal slice. Fluorescent channels: GCaMP7f (green) and hM3Dq-mCherry (red); outlines: BNST (blue), insula (orange), and fiber track (white). **d**, **e** Respective injection sites. **d** Right BNST. **e** Right insula. **f** BNST calcium transient time-locked to struggling onset. (CNO = gray, saline = blue). **g** Area under the curve (AUC) of *Z* scores from all bouts of CNO- (gray) and saline (blue)-treated animals. Welch two-sampled *t* test, *t*_(494.56)_ = 3.1668, **P = 0.0016. **h** Average maximum peak *Z* score from all bouts of CNO- (gray) and saline (blue)-treated animals. Welch two-sampled *t* test, *t*_(467.1)_ = 3.9858, ***P = 0.0008. **g**, **h** CNO: n = 813 bouts from five animals. Saline: *n* = 910 bouts from six animals. **i** Diagram of light–dark box with three zones (light, center, and dark). Mice were placed in the center chamber of the light–dark box for 10 min following restraint testing. **j** Percent of total time spent in each zone (light, center, and dark) by CNO- (*n* = 5) and saline (*n* = 5)-treated animals. Multiple unpaired *t* tests with the false discovery rate (FSD) approach of two-stage step-up method of Benjamini, Krieger, and Yekutieli. FSD = 1%. light zone: *t*_(8)_ = 1.833, *P* = 0.104; center zone: *t*_(8)_ = 1.463, *P* = 0.1814; dark zone: *t*_(8)_ = 2.636, *P = 0.0299. **k** BNST calcium transient frequency in each zone (light, center, and dark) by CNO- (*n* = 5) and saline (*n* = 4)-treated animals. Multiple unpaired *t* tests with the FSD approach of two-stage step-up method of Benjamini, Krieger, and Yekutieli. FSD = 1%. light zone: *t*_(7)_ = 1.660, *P* = 0.1410; center zone: *t*_(7)_ = 0.0965, *P* = 0.9258; dark zone: *t*_(7)_ = 1.255, *P* = 0.2499. **l** Average BNST calcium transient amplitude in each zone (light, center, and dark) by CNO- (*n* = 5) and saline (*n* = 4)-treated animals. Multiple unpaired *t* tests with the FSD approach of two-stage step-up method of Benjamini, Krieger, and Yekutieli. FSD = 1%. light zone: *t*_(7)_ = 2.096, *P* = 0.0743; center zone: *t*_(7)_ = 0.9764, *P* = 0.3614; dark zone: *t*_(7)_ = 2.527, *P = 0.0394.

After demonstrating that insula G_i_-DREADD activation decreased BNST *fos* following restraint, we tested the hypothesis that specifically activating insula^→BNST^ neurons could change BNST activity during restraint. We injected a 1:1 ratio of AAV9.hSyn.GCaMP7f and AAVrg-Cre (retrograde AAV-expressing Cre) into the BNST and Cre-dependent G_q_-DREADD (hM3Dq) into the insula. CNO (i.p. 3 mg/kg) injection then selectively activated the G_q_-DREADD in insula^→BNST^ neurons, while calcium transients from the BNST were recorded during restraint (Fig. 5a–e). Averaged time-locked bout-associated transients can be seen in Fig. 5f. CNO-treated animals exhibited higher average BNST peak transient amplitudes and AUCs from 0 to 5 s after bout onset (Fig. 5g, h). Interestingly, G_q_-DREADD activation did not significantly change the characteristics of the bouts themselves, including the number and duration of bouts and total struggle time (Supplementary Fig. 8). We hypothesized that G_q_-DREADD activation of insular input to the BNST would exacerbate post-stress avoidance of the light side in the light–dark box, a test validated for assessing negative affective state in mice^30^, immediately after restraint (Fig. 5i). Indeed, CNO-treated mice spent significantly more time in the dark side than saline-treated mice (Fig. 5j), suggesting a DREADD-induced increase in negative affective behavior. BNST signaling also changed with CNO, such that treated animals exhibited higher average GCaMP transient amplitude (but not frequency) in the dark zone and a trend toward higher amplitude in the light zone (Fig. 5k, l). We next performed the same experiment in mice using a pan-neuronal G_i_-DREADD in bilateral mid-insular neurons. Inactivating mid-insular neurons decreased BNST GCaMP transient amplitude at the onset of struggling bout behavior during restraint stress (Supplementary Fig. 9), but unlike activating insula^→BNST^ neurons, inactivating mid-insula neurons did not change subsequent behavior in the light–dark box (*F*_(1,13)_ = 0.114, *P* = 0.741).

### The afferent network for the insula^→BNST^ pathway is broadly distributed and includes significant somatomotor input

**Fig. 6:**
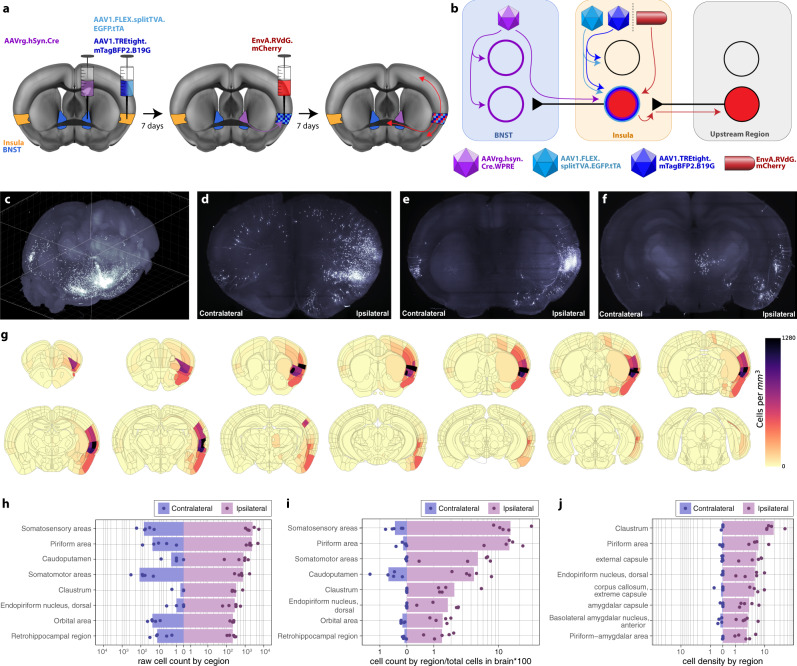
The control network for the insula^→BNST^ pathway includes piriform, somatosensory, somatomotor, and amygdalar nuclei. **a** Diagram of the viral strategy for tracing the control network for insula^→BNST^ cells. AAVrg.Cre (purple) was injected into the BNST. Viruses carrying the Cre-dependent helper proteins TVA (cyan) and B19G (blue) were injected into the insula. After seven days, rabies virus (EnvA.RVdG.mCherry; red) was injected into the same region of the insula. Seven days later, the brains were collected and started on the light sheet microscopy pipeline. **b** Schematic of rabies tracing strategy. AAVrg.Cre infects cells locally in the BNST and afferents. The TVA and B19G viruses infect many neurons near the injection site, but only express their proteins in insula^→BNST^ neurons that also express Cre. Entry of the modified rabies is restricted to TVA-expressing cells, and transsynaptic retrograde infection is limited to cells expressing B19G. Therefore, labeled neurons outside of the injection region are monosynaptically connected to the insula^→BNST^ neurons. **c** Maximum projection image of 3D reconstruction of light sheet data set to illustrate insula^→BNST^ control network. Olfactory bulbs are in the bottom left corner, and the cerebellum is in the top right corner. **d** Resampled images in the coronal plane at the level of the anterior insula. **e** At the level of the BNST, and **f** at the level of thalamic and amygdalar structures. **g** Distribution of labeled neurons represented as a composite whole-brain density heatmap (averaged from five mice) of semiautonomously quantified insula^→BNST^ control network cells as density in mm^3^. **h** Graphical representation of cell densities in top eight regions (by raw cell count) labeled as part of the labeled insula^→BNST^ control network of five mice. Bars: mean from all three counts per region. Points: counts from individual animals. Ipsilateral counts are on the right in purple. Contralateral counts are on the left in blue. The *x*-axis is an inverse hyperbolic sine transformation. **i** Top eight regions (by cell count per region/total cells in each respective brain × 100) of the labeled insula^→BNST^ control network. **j** Top eight regions (by cell density) of the labeled insula^→BNST^ control network.

Struggling bouts are tightly coupled with insula–BNST signaling, yet it is unclear how motor information reaches this pathway. In an effort to understand the control network for the insula^→BNST^ pathway, we turned to a rabies-based tracing method, TRIO (tracing the relationships between inputs and outputs)^31^, to selectively label the inputs directly synapsing onto insula^→BNST^ neurons (Fig. 6a–f, Supplementary Fig. 10a–f, and Supplementary Movie 5). Five brains were registered with the Allen Brain Atlas Coordinate Reference Framework^32^. The densities of labeled neurons in each region were quantified, averaged across the five samples and the results overlayed on the Allen Brain Atlas (Fig. 6g and Supplementary Fig. 10a, b). Raw cell counts and normalized cell counts (normalized to the total number of neurons in respective brains) revealed high densities of afferent neurons in somatosensory, piriform, and somatomotor regions (Fig. 6h, i, Supplementary Fig. 11a, and Supplementary Movies 6 and 7). Relative to the overall volume of certain structures, high densities of labeled cells were found in smaller regions, such as the claustrum and basolateral amygdala (Fig. 6j and Supplementary Fig. 11b). Because the TRIO findings detailed a motor component that had yet to be explored, we evaluated this pathway’s fidelity using other convergent viral techniques. Indeed, the TRIO data were compatible with retrogradely labeled motor input to the mid-insula (Supplementary Fig. 12a–h, and Supplementary Movies 8–10), and the distribution of anterogradely labeled cells receiving input from the motor cortex (Supplementary Fig. 12i–n and Supplementary Movie 11).

**Fig. 7:**
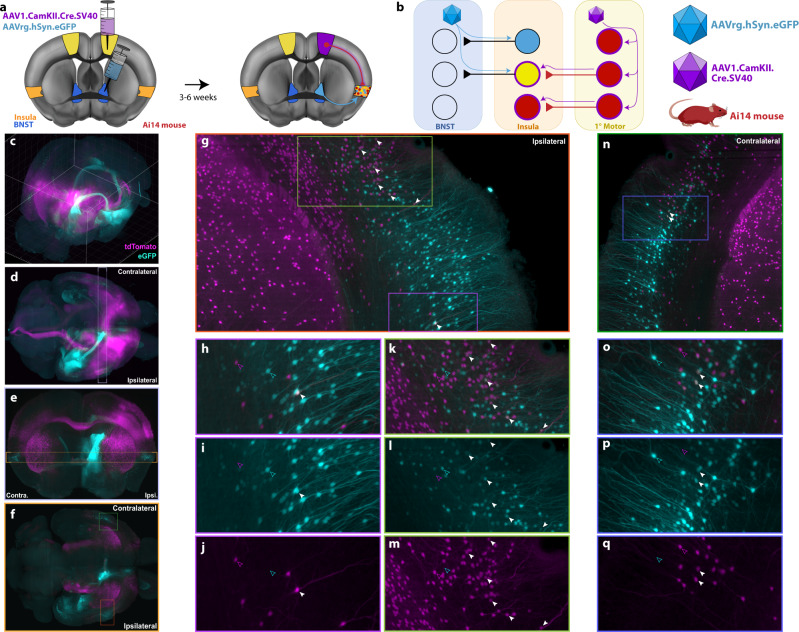
Motor efferents project onto insula^→BNST^ cells. **a** Diagram of AAVrg.eGFP (cyan) injected into the BNST (blue) and AAV1.Cre (purple) injected into motor cortex (yellow) of Ai14 mice. **b** Schematic of the viral strategy. AAVrg.eGFP retrogradely labeled insula^→BNST^ cells. AAV1.Cre labeled neurons that are downstream from the motor cortex. Neurons that were downstream from motor cortex and projected to the BNST were doubly labeled (yellow). **c** Maximum projection image for 3D reconstruction of whole-brain labeling; eGFP shown in cyan and tdTomato shown in magenta. Olfactory bulbs are in the bottom left corner, and the cerebellum is in the top right corner. **d** Top-down view of whole brain. Light purple outline indicates the slice taken for the next image. Olfactory bulbs are to the right. Cerebellum is to the left. **e** Coronal slice taken at the level of the BNST. Yellow line indicates the transverse slice taken for the following image. **f** Maximum intensity projection of a transverse slice taken at the level of the insula. Dark green and orange boxes indicate regions of the insula that project to the BNST. They are expanded later in the figure. **g** Magnification of orange box from **f**. Image is ipsilateral to the injection site. Light green and purple boxes are expanded and split by channel in later images. White arrowheads indicate doubly labeled neurons. **h** Magnified image of purple box from **g**. Solid white arrowheads indicate doubly labeled neurons. Cyan open arrowheads indicate eGFP-only labeling. Magenta open arrowheads indicate tdTomato-only labeling. **i** eGFP-only channel from **h**. tdTomato signal does not bleed through (see open magenta arrowhead). **j** tdTomato-only channel from **h**. eGFP signal does not bleed through (see open cyan arrowhead). **k** Magnified image of light green box from **g**. **l** eGFP-only channel from **k**. **m** tdTomato-only channel from **k**. **n** Magnification of dark green box from **f**. Image is contralateral to the injection site. Blue box is expanded and split by channel in later images. **o** Magnified image of blue box from **n**. **p** eGFP-only channel from **o**. **q** tdTomato-only channel from **o**.

We next aimed to isolate the specific insula^→BNST^ neurons that receive motor input. We injected an anterograde transneuronal Cre virus (AAV1.Cre) into the motor cortex of Ai14 (Cre-dependent tdTomato expressing) mice and retrograde eGFP virus (AAVrg.eGFP) into the BNST (Fig. 7a). This strategy labeled neurons that send projections to the BNST with eGFP, while labeling cells that receive inputs from the motor cortex with tdTomato (Fig. 7b). We observed largely discrete populations of neurons labeled with either eGFP or tdTomato (Fig. 7c–f and Supplementary Movies 12). However, in the insula, eGFP (+) cells occupied space that overlapped with the tdTomato (+) cell distribution. The tdTomato cells dominated rostrally, becoming fewer in number relative to those labeled with eGFP at more caudal levels. Doubly labeled cells (Fig. 7g–m) were sparse but reproducibly found throughout the insula. This also held true contralaterally (Fig. 7n–q). Doubly labeled cells were readily distinguished from singly labeled cells, providing further distributional information about the location of the diffuse insular neurons involved in the ^motor→^insula^→BNST^ pathway.

**Fig. 8:**
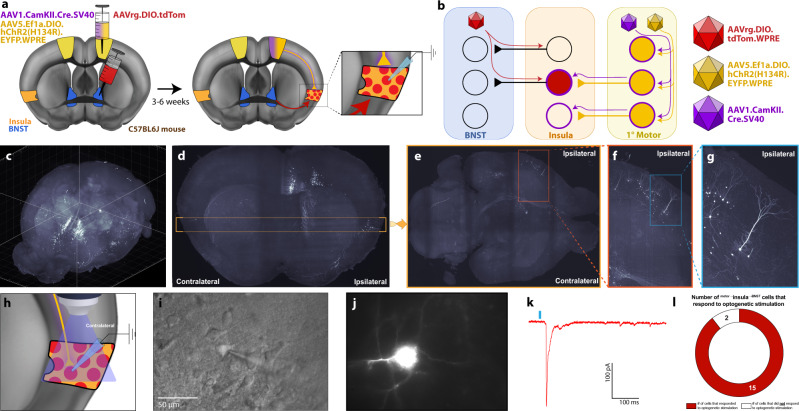
Motor efferents directly synapse onto and activate insula^→BNST^ cells. **a** Diagram of the viral injections to isolate the ^1°Motor→^insula^→BNST^ cells. A 1:1 ratio of AAV1.Cre (purple) and AAV5.DIO.ChR2.EYFP (yellow-green) were injected into the motor cortex. AAVrg.DIO.tdTomato (red) was injected into the BNST. We then performed whole-cell patch-clamp recordings on the labeled cells. **b** Schematic of viral labeling. AAVrg.DIO.tdTomato infects cells that project to the BNST, but will only cause the expression of tdTomato in the presence of Cre. AAV1.Cre will infect neurons that are downstream from the motor cortex. Thus, only neurons that are both downstream of the motor cortex and send projections to the BNST are labeled. ChR2 is expressed in neurons in the motor cortex and their efferents. **c** Maximum projection image for 3D reconstruction of whole-brain labeling to illustrate ^1°Motor→^insula^→BNST^ cells. **d** Resampled image in coronal plane at the level of the BNST. Yellow box indicates the transverse slice taken for the following image. **e** Resampled image in transverse plane through the insula. Orange box is magnified in the next image. **f** Magnified view of orange box (ipsilateral to the injection site) in **e**. Light blue box is magnified in next image. **g** Magnified view of light blue box in **f**. Examples of ^1°Motor→^insula^→BNST^ cells. **h** Diagram of whole-cell patch-clamp experiment to verify motor input onto presumptive ^1°Motor→^insula^→BNST^ cells. **i** DIC image with pipette next to insula neuron. **j** Example of fluorescently labeled ^1°Motor→^insula^→BNST^ cells visible on the electrophysiology rig. **k** Example of optically evoked postsynaptic current after blue light stimulation (blue bar). **l** Number of patched tdTomato-positive cells that responded to light (*n* = 17 cells from five animals).

Next, we aimed to determine if motor cortex afferents could drive insula^→BNST^ neurons (i.e., if the ^motor→^insula^→BNST^ represents a functional synapse). We used alternative viral strategies to confirm the doubly labeled cells’ identity and then provide efficient visual identification for whole-cell patch clamp. We injected AAV1.Cre into the motor cortex and a Cre-dependent AAVrg.DIO.tdTomato into the BNST before processing the tissue for whole-brain imaging (Fig. 8a, b). Consistent with our findings above, light sheet microscopy revealed specific insular distributions most densely distributed in the mid and posterior insula, demarcating a circuit from ^1°Motor→^insula^→BNST^ (Fig. 8c–g). Finally, to verify monosynaptic connectivity and functionality of this synapse, we performed channelrhodopsin-assisted circuit mapping (CRACM)^33^ with whole-cell patch clamp in similarly tagged insula cells, while stimulating motor terminals (Fig. 8h, i). We injected channelrhodopsin into the primary motor cortex and recorded whole-cell currents from insula^→BNST^ cells (tdTomato-positive cells). Blue light stimulation resulted in a reliable optically evoked excitatory postsynaptic current (oEPSC) in 15 of the 17 (or ~88%) labeled cells we patched (Fig. 8k, l). This suggests that the labeled cells, which project from the insula to the BNST, receive functional input from cortical motor neurons.

### Primary motor cortex neurons projecting to the mid-insula (motor^→insula^) increase signaling prior to the initiation of a struggle bout

**Fig. 9:**
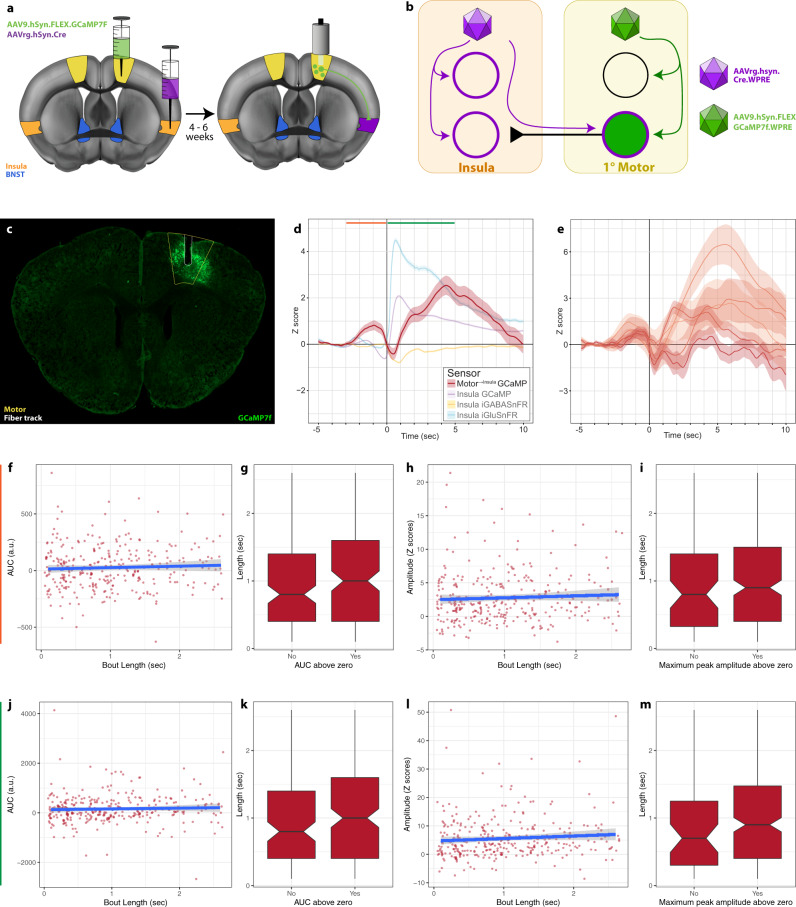
1°Motor^→insula^ cells are engaged during struggling. **a**, **b** Schematics. **c** Representative coronal slice. Fluorescent channel: GCaMP7f (green); lines: 1°Motor cortex (yellow) and fiber track (white). **d** Average of all bout-associated signals from GCaMP in 1°Motor^→insula^ cells (red). Insular recordings from Fig. 4e included as faded colors for comparison. Insular GCaMP (purple), SF-iGluSnFR (blue), or iGABASnFR (yellow). Lighter shading: s.e.m. across bouts. Green and orange lines at the top of graph indicate the corresponding windows for analysis. A total of −3 to 0 s preceding bout onset (orange) corresponds to **f**–**i**. A total of 0–5 s following bout onset (green) corresponds to **j**–**m**. **e** Bout-associated signals separated by animal. **f**–**i** Analysis of 1°Motor^→insula^ GCaMP during window from −3 to 0 s preceding bout onset. Blue line: slope with 95% confidence interval. **f** AUC vs. bout length (s). Pearson correlation: *r*_(307)_ = 0.047, *P* = 0.412. **g** Bout length of bouts with an AUC > 0 (“yes” = >0, *n* = 164 from five animals) vs. those with an AUC not >0 (“no” = not >0, *n* = 145 from five animals). Welch two-sampled *t* test, *t*_(307)_ = −1.6177, *P* = 0.1068. **h** Maximum peak *Z* score amplitude vs. bout length (sec). Pearson correlation: *r*_(307)_ = 0.0515, *P* = 0.366. **i** Bout length of bouts with a maximum peak > 0 (*n* = 239 from five animals) vs. those with a maximum peak not >0 (*n* = 70 from five animals). Welch two-sampled *t* test, *t*_(119.69)_ = −1.6229, *P* = 0.1072. **j**–**m** Analysis of 1°Motor^→insula^ GCaMP during window from 0 to 5 s following bout onset. **j** AUC vs. bout length (s). Pearson correlation: *r*_(307)_ = 0.036, *P* = 0.5285. **k** Bout length of bouts with an AUC > 0 (*n* = 187 from five animals) vs. those with an AUC not >0 (*n* = 122 from five animals). Welch two-sampled *t* test, *t*_(266.15)_ = −1.5977, *P* = 0.1113. **l** Maximum peak *Z* score amplitude vs. bout length (s). Pearson correlation: *r*_(307)_ = 0.086, *P* = 0.157. **m** Bout length of bouts with a maximum peak > 0 (*n* = 258 from five animals) vs. those with a maximum peak not >0 (*n* = 51 from five animals). Welch two-sampled *t* test, *t*_(72.93)_ = −1.5534, *P* = 0.1247. **g**, **h**, **k**, **m** Median, quartiles, and 1.5 × the interquartile range are indicated with the associated box plot.

Our findings suggest somatomotor projections to the insula can functionally impact insula neuron signaling. However, the contribution of insula-projecting motor cortex neurons to struggling behavior is unknown. To investigate this further, we isolated motor^→insula^ neurons by virally transducing mid-insula neurons with retrograde AAVrg.Cre and injecting Cre-dependent GCaMP7f into the primary motor cortex (1°Motor). We then placed a fiberoptic implant in the motor cortex (Fig. 9a–c). After 4–6 weeks, mice were subjected to restraint stress while simultaneous GCaMP measurements were collected. We detected a distinct signaling pattern from those observed in the insula and BNST, as an increase in calcium signaling in motor^→insula^ cells preceded the bout onset (Fig. 9d, e). The signal then returned to baseline before significantly increasing again shortly after the bout onset. As opposed to the insula signals, the maximum peak occurred several seconds after the struggle bout onset (Fig. 9d, e). Further, there was no correlation between bout length and AUC or maximum peak amplitude (Fig. 9f–m). Thus, motor^→insula^ cells represent a distinct population of neurons recruited both prior to and during motor action.

## Discussion

Effective stress-coping strategies are critical for survival, yet how coping strategy participation is encoded across neuronal circuits is not well understood. Here, we delineated a circuit involved in affective behavior that encodes active struggling during restraint stress, a potential correlate to active stress coping in mice. In this study, we delineate a mid-insula→BNST^CRF^ neuron circuit that is recruited in tight association with struggling events. Even though this circuit’s activity closely followed struggling behavior, chemogenetically manipulating this circuit did not alter struggling during restraint. It did, however, change post-stress avoidance after the stressor, indicating this pathway encodes aspects of stress coping or its resultant outcome.

We also explored the relationship between insular signaling and struggling duration during repeated restraint stress. While a correlation between extracellular glutamate signal size and bout length remained stable over time, this relationship collapsed with repeated homotypic stress for both inhibitory and local calcium signals. This divergence may indicate that plasticity during homotypic stress occurs downstream of the glutamate afferents into the insula.

To begin to assess plausible neuronal paths for incoming information to reach this circuit, we generated extensive input maps for the insula^→BNST^ control network and identified prominent afferent regions. These findings show tightly correlated struggling-related activity in the cortical insula and subcortical BNST, along with an upstream network that includes significant motor, somatosensory, and amygdalar components. Given that the struggling bout is a motoric program, we chose to investigate the large motor input, characterizing the motor^→insula^ pathway’s activity at struggling onset. We discovered that its calcium activity slightly preceded behavior initiation. Thus, we hypothesize this input may be a mechanism by which executive motor control circuits can inform interoceptive and affective circuits.

### BNST ^CRF^ neurons encode active struggling behavior during an inescapable stressor

Distinct subnuclei of the BNST process sustained fear states and the capacity to respond to threats via a network of topographically organized connections that extend throughout the brain^27,34^. CRF neurons in the BNST have been implicated in reinforcement/consumptive behaviors, and appear to play a role in regulating negative affective state^35–37^. Our findings demonstrate that these cells monitor struggling responses. Consistent with the previous work implicating BNST^CRF^ cells in stress responses^22^, we show that increased BNST^CRF^ cell calcium transient signals are robustly time-locked to the onset of struggling bouts (Fig. 3f–h). Struggle bout-associated glutamate transients in the BNST suggest a circuit dependence to these signals (Fig. 3i–k). Together, these findings are consistent with Lebow et al.’s proposed “valence surveillance” role for the BNST, in which this region integrates internal mood and arousal with external contexts to coordinate fight-or flight via the HPA axis^27^. Our work expands this view to incorporate monitoring the impact of motor action on a subject’s state. While the direct correlation of struggling behavior to stress coping in mice remains to be explored, the relationship between the struggling bouts and activity in BNST and BNST-connected circuits suggests this behavior is at least partly related to larger coping mechanisms. Consequently, we propose that the BNST may be involved in a larger network surveilling struggling actions, when exposed to an inescapable stressor.

### The insula sends a dense, unidirectional input to the lateral BNST (insula^→BNST^) that serves as a conduit for encoding struggling behavior information and alters BNST function and affective behavior

Gogolla^6^ proposed that the variety of afferents (e.g., sensory, limbic, autonomic, and frontal) converging in the insula may be a setting for cross-functional association. The distribution of inputs that we found to be retrogradely labeled from our injection site in the insula (Supplementary Fig. 4a–i) bolsters the idea that the insula may indeed link internal state with external cues. There is also evidence that in rodents, the mid-insula is involved in valence computation^38^. Thus, the insula could be a critical node for the coalescing of state information, assigning it a valence, and passing it on to relevant network structures to engage the most appropriate response.

Recent human studies suggest insular–BNST interactions may be important across species^39^. Here, we identified the region of the mouse insula with the densest projection to the BNST (near AP = 0.0), and chemogenetically inhibited this area to determine its role in modulating the stress-responsive BNST^CRF^ cells. Indeed, decreasing mid-insular activity with inhibitory G_i_-DREADD decreased restraint-induced *fos* in BNST^CRF^ neurons, indicating insular inputs can modulate the engagement of these cells during a stressor (Fig. 4a–c). This is in agreement with our previous work, demonstrating that insular G_i_-DREADD activation can decrease excitatory drive onto BNST cells^22^. To interrogate the insular signaling responsible for this change, we used in vivo fiber photometry to record insular signaling during restraint. As in the BNST, insular calcium signals were tightly associated with struggling behavior in the RESTRAINT device (Fig. 4d–f). This insular calcium transient was paralleled by a tightly coupled decrease in GABA release and increased glutamate release (Fig. 4d–f), suggesting that altered excitatory–inhibitory balance leads to insular recruitment of BNST. Interestingly, bout duration was positively correlated with insula GCaMP and SF-iGluSnFR signal and negatively correlated with iGABASnFR signal. Repeated exposure to the homotypic stressor resulted in decreased correlation with GCaMP and iGABASnFR, but not SF-iGluSnFR signal providing intriguing mechanistic insight into the relationship between stress habituation and insular microcircuitry. Further, rimonabant restored the correlation for GCaMP but not iGABASnFR, suggesting rimonabant-induced anxiogenesis is not mediated through inhibition of insular GABA signaling.

We additionally assessed real-time changes in struggle event-associated calcium transients in the BNST by manipulating the insular input. G_q_-DREADD, specifically targeted to insula^→BNST^ cells, increased the mean peak amplitude of BNST active struggle event-associated calcium signals following CNO administration (Fig. 5a–h). Activating this subset of cells also increased subsequent anxiety-like behavior and BNST calcium transient frequency during behavioral testing (Fig. 5i–l). Taken together, we propose that these findings indicate that the struggle response engages the insula^→BNST^ pathway.

### The insula^→BNST^ control network reveals significant motor and premotor cortical input

Our initial brain-wide retrograde AAV tracing experiment demonstrated a dense insular projection to the BNST (Supplementary Fig. 4a–i). Later, in order to determine paths by which motor regions might transmit information to the insula, we used a retrograde viral tracing strategy to label cells across the brain from the insular region that projected most densely to the BNST (Supplementary Fig. 12a–h). This strategy was not specific to the inputs of insula^→BNST^ neurons. Next, using a whole-brain imaging approach and monosynaptic rabies tracing, we created a global map of the upstream control network for the insula^→BNST^ pathway specifically. The rabies virus labeled cells in multiple brain regions, including many regions anticipated from previous traditional tracer studies, such as somatosensory cortex and parafascicular nucleus of the thalamus^40^. Moreover, neurons in the premotor and motor cortex were heavily represented.

As a convergent anatomical strategy, we combined retrograde (from the BNST) and anterograde (from the 1°Motor) viruses, and identified insula^→BNST^ neurons that receive inputs from 1°Motor cortex. We later confirmed functional connectivity of this synapse using channelrhodopsin-assisted mapping and whole-cell patch-clamp electrophysiology (Fig. 8). Doubly labeled cells were sparse yet consistently found bilaterally in the insula, providing evidence of direct motor input onto affective state control circuitry (Fig. 7g–q). A far greater proportion of cells were infected with only one virus, yet, when compared with other regions, these two populations of singly infected cells appeared to be intermixed in the insula to a greater degree. This could be evidence of an avenue for polysynaptic modulation of an affective circuit by the motor cortex. Of note, while we focused on insular cells that project to the BNST, our results do not imply that insula^→BNST^ cells are the sole subpopulation receiving the identified inputs. Instead, we suspect it is more likely that the regional weights of the upstream distribution would differ based on the selected insular output or subpopulation. Previous insular mapping studies demonstrate distinct distributions of inputs to insular neurons, and moreover, the various outputs of insular neurons are largely discrete^41,42^. This suggests that monosynaptic tracing studies with different insula starter populations could yield upstream circuit maps involved in varying insula-related behaviors.

It bears noting that AAV1.Cre viral labeling is known to have retrograde properties, which can complicate interpretation when used in a bidirectional circuit^43,44^. The insula^→BNST^ pathway appears unidirectional (Fig. 7d)^41^, thus greatly limiting spurious labeling. However, we cannot rule out this possibility for insula^→1°Motor^ cells. We used numerous convergent techniques to mitigate that potential ambiguity. In addition, AAV1.Cre labeling may be activity dependent^44^. If this is indeed the case, low-activity or silent synapses could be falsely omitted. However, it is unlikely that this dependency would lead to false-positive cells; rather, undersampling the cells that receive input is a more likely scenario.

### Motor pathways to BNST-projecting insular neurons reveal a path for motor planning information to potentially influence affective circuitry

This study provides the first evidence for a functional pathway by which affective circuitry can be directly informed of motor activity by structures involved in motor planning. While the BNST, central to longitudinal threat evaluation, receives no detectable direct motor input based on our tracing studies, the insula receives strong, broadly somatotopically distributed input from cortical motor regions. While a number of studies have outlined the role of physical activity in the regulation of affective behavior, they have focused on feedback mechanisms by which peripheral metabolites and other peripheral signals may convey information to the CNS to alter mood^45–48^. The present work identifies a feedforward pathway by which information relevant to physical activity can regulate affective circuitry directly, and we hypothesize that this may be consistent with the concept of efference copy that has been applied in other fields.

Efference copy is defined as collateralized information that works in parallel to the signals directly involved in motor action. This is a mechanism by which the brain can compare predicted actions to resultant outcomes and sensations^49^. The ability to maintain an undisturbed visual field during saccades and the inability to tickle oneself are functional examples of motor efference copy^50–52^. Thus, we propose that the motor^→insula^ pathway may represent an efference copy through which the insula^→BNST^ pathway acts as a motor surveillance system. The motor projections to the insula^→BNST^ may relay predicted motor action outcomes. In our restraint model, the discrepancy between the anticipated outcome from struggling, and the reality of remaining restrained may generate and maintain the negative affective state associated with an inescapable stressor, driving increased activity in the insula and BNST.

Consistent with an efference copy model, G_q_-DREADD activation of insula^→BNST^ neurons did not alter the number of struggle bouts (but did alter BNST activity), nor did the inactivation of mid-insula with a G_i_-coupled DREADD (Supplementary Fig. 9a–j). These results could suggest a model through which struggling behavior and affective state are basally correlated as a result of efference copy. In this theory, chemogenetically altering insula^→BNST^ activity could dissociate struggling behavior from affective state, resulting in the altered affect without impacting struggling behavior. To our knowledge, this is the first model to incorporate an affective/interoceptive circuit into the efference copy theory. While further studies are needed to directly test this idea, this foundational work may help pave the way for future convergent studies exploring the involvement of feedforward motor pathways, contributing to situational affective state.

Notably, increased activity in motor^→insula^ neurons appeared to be left-shifted compared to struggling behavior onset and average insular glutamate and calcium transients. Because we hypothesize the motor input communicates movement planning/initiation, this signal preceding movement would be predicted. The motor^→insula^ signals were recorded in motor cortex, while the other recordings were conducted in the insula or BNST, thus confounding true temporal comparisons between these transients. It should be noted that motor innervation is not the only glutamatergic afferent for this circuit, and that other inputs may also play important roles in driving this activity. Indeed, we found that the amygdala sends a strong input to insula^→BNST^ cells, and moreover, the density of somatosensory input onto these neurons was greater than that of the motor input. We suspect it is likely that these and other regions participate in regulating the insula^→BNST^ pathway by passing other relevant data. For example, it is conceivable that sensory and affective information feed into the insula^→BNST^ pathway via the somatosensory cortex and amygdala, respectively. In contrast to the BNST, however, our AAV1-Cre data indicate that the motor cortex connects densely with both of these regions, thus information shaped by motor commands may also reach the insula^→BNST^ circuitry through polysynaptic connections in other brain areas. More studies will be needed to fully elucidate the breadth of functions held by the insula^→BNST^ pathway, and its afferent and efferent networks. In all, this work provides foundational evidence that the insula^→BNST^ pathway is part of a large and diverse system governing affect following a stressor, and we anticipate ensuing work will determine the distinct roles of relevant inputs to this pathway.

## Methods

All reagents and resources used in this paper can be found in Supplementary Table 1. Full primer sequences for transgenic mice used in this paper are listed in Supplementary Table 2.

### Animals

The mice used in this study were female and at least 8 weeks old. C57BL/6J mice (RRID:IMSR_JAX:000664; The Jackson Laboratory) were delivered at 6 weeks of age and acclimated for 2 weeks prior to intervention. Crh-IRES-Cre (RRID:IMSR_JAX: 012704; The Jackson Laboratory) and PKCδ-Cre mice (RRID:MMRRC_011559-UCD; Mutant Mouse Resource and Research Centers) were bred and genotyped in-house to be heterozygotes^53^. Homozygous Ai14 (RRID:IMSR_JAX: 007908; The Jackson Laboratory) mice were purchased and bred in-house, and maintained as homozygotes therefore genotyping was not used^54^. Transgenic lines were maintained on a C57BL/6J background. All mice were housed with two to five mice per cage and provided food and water ad libitum. Light/dark cycle was 12 h (lights on = 0600 h) with controlled humidity (30–50%) and temperature (20–25 °C). Behavioral testing and surgeries were conducted during the light phase. The Vanderbilt Animal Care and Use Committee approved procedures and interventions.

### Reagents

CNO (Sigma-Aldrich #C0832, > 98%;) was diluted in sterile saline (3 mg/10 ml = 875 µM) and delivered intraperitoneally at a dose of 3 mg/kg.

AAVs were used as received unless otherwise specified. TREtight-mTagBFP2-B19G and syn-FLEX-splitTVA-EGFP-tTA were diluted 1:20 and 1:200, respecitvely, in sterile, filtered PBS, and subsequently mixed 50/50 by volume.

### Stereotaxic surgeries

Mice (>8 weeks) were anesthetized with isoflurane (3% initial dose, 1.5% maintenance dose) for intracranial recombinant AAV injection and fiberoptic cannula implant surgeries, using a Leica Angle Two Small Animal Stereotaxic Instrument. Mice were injected with 300 nL of the indicated AAV at a rate of 50 nL/min driven by a Micro4 MicroSyringe pump (World Precision Instruments) into the specified region. The needle (World Precision Instruments Nanofil syringe fitted with a Nanofil 33 G Blunt Needle) remained in place for an additional 5 min. All mice recovered for at least 3 weeks before further experimentation. Injection and implantation sites included insular cortex (from Bregma: AP = 0.02, ML ± 3.66, DV = −4.30, 0° angle), dorsal BNST (from Bregma: AP = 0.14, ML ± 0.88, DV = −4.24, 15.03° angle), and primary motor cortex (from Bregma: AP = 1.60, ML ± 1.10, DV = −1.80, 15.03° angle). All mice received 5 mg/kg injections of meloxicam for once a day for 2 days following surgery.

#### Chronic optical fiber implantation specific procedures

Once the skull was exposed, it was etched with a gel etchant (Kerr Dental). One stainless-steel mounting screw (PlasticsOne) was installed in the ipsilateral parietal plate posterior to the implant hole. The implant was bonded by applying Optibond primer followed by Optibond adhesive, which was then cured with UV light. Herculite enamel was molded around the screw, implant, and skull. The enamel was then cured with UV light.

Fiberoptic implants for the ChiSquared Bioimaging system were constructed in-house using a 0.22 NA, 300 μm core multimode fiber (Thorlabs). Fibers were cut to length using a DualScribe Wedge Tip Carbide Scribe (Ideal Industries), and secured inside a mounting ferrule with Epo-Tek general room temperature cure epoxy (Fiber Optic Center) and cured at 45 °C overnight. The end to remain exposed was then cut to be flush with the implant and polished using a ferrule polishing disk (Thorlabs) and progressively finer (5, 3, 1, and 0.1 μm) aluminum oxide lapping sheets (Thorlabs). Fibers were tested prior to implantation to ensure >90% power output. Mono fiberoptic cannulas (Doric Lenses) for the Tucker-Davis Technologies (TDT) system were purchased at the appropriate lengths for each brain region.

### Restraint stress

#### RESTRAINT device

The base was made from clear acrylic (McMaster-Carr). The screw and rear-guard components were made from extra plastic from our machine shop (Vanderbilt Kennedy Center Scientific Instrumentation Core). See diagram for detailed dimensions (Supplementary Fig. 1a–e).

Fibers were attached to each animal’s implant with a stainless-steel sleeve (inner diameter = 400 µm) before the animal was placed inside the restraint device. Restraint exposure lasted 30 or 60 min. Video was captured with a webcam (Logitech), and movements were tracked using DLC.

#### Drug delivery

CNO was administered i.p. 30 min prior to restraint at a dose of 3 mg/kg. Equal volume saline was administered to the vehicle groups.

### Behavioral scoring

All videos were acquired at 10 fps using a Logitech C920 camera. For experiments detailed in Figs. 1d, e and 3i–k, behavioral scoring was done manually using Anvil 6.0 video annotation software. Behavioral bouts were counted if the tail head and tail movement were visible on the recording. Bouts were considered contiguous if they were separated by <0.7 s. Automated video scoring was performed using DLC to track points across time and was based on the methods used for manual scoring^14,15^. The fiberoptic cable and the tip of the tail were manually located in each image of a training set made up of >15 images per video. A small green piece of tape was placed on the fiberoptic cable to ensure a consistent location during training and testing. DLC was trained for at least 200,000 iterations. We used R statistical software with the “tidyverse” package to convert X/Y position into speed of movement for the fiber and the tail during each frame^55,56^. To identify bouts, we first identified frames in which the fiber and tail were moving. We determined if the tracked objects were moving by setting speed thresholds (selected via trial and error) that were independently set for both the fiber and the tail tip. For the fiber, any frame with a speed greater than one standard deviation above the bottom 95% of frame speeds was considered mobile. For the tail tip, the threshold was set at three standard deviations above the bottom 99% of tail tip frame speeds. The use of a normalized speed, rather than a raw pixel-based speed threshold, provided better consistency across trials because it accommodated slight changes in camera distance and restraint device position. When a tracked point transitioned from an immobile to a mobile state, that frame became time 0.0 for a bout. The bout continued until all tracked points were in an immobile state for >0.7 s (to limit significant bout overlap). We observed three bout types: (1) movements of the head only, (2) movements of the tail only, and (3) movements that included both head and tail movements, termed full body. Unless otherwise noted, we specifically focused our analysis on full body movements. To make the analysis uniform across bouts, AUC, and maximum peak amplitude were calculated for the 5 s, following bout onset independent of bout length. We used R with the pheatmap package to create the time-locked photometry heatmaps^57^. Figures were generated in R with the ggplot2 package^58^.

### Wheel running

#### Training

One week prior to testing, we placed a low-profile running wheel (Med Associates) in the home cages of the mice to be tested, for 48 h. These wheels use the Fast Track 100% PETE plastic wheel surface from Bio-Serve (K3250). The wheel was removed for the 5 days preceding testing.

#### Testing

Animals with fiberoptic implants had free access to the same style of low-profile running wheel for 30 min in their home cage. Running behavioral scoring was done manually using Anvil 6.0 video annotation software. Running was defined as being both on the wheel and running (i.e., not standing on the wheel nor swinging back and forth on the wheel were counted).

### In vivo fiber photometry

The ChiSquare *χ*^2^−202 System (ChiSquare Biomaging) was one fiber photometry system used in this study. Briefly, blue light from a 473-nm picosecond-pulsed laser (50 MHz; Becker & Hickl) was directed onto a GFP dichroic filter (MD498, Thorlabs) and coupled using a FC/PC fiber coupler (Lasos) into a multimode fiber patch cord (FG105UCA, 0.22 N.A., 105/125 µm diameter core/cladding, Thorlabs) terminating in the ferrule described above. Fluorescence emission from the tissue was collected through the same fiber transmitting the blue light using a fiber coupler (Thorlabs) into a 200 µm diameter core, antireflection-coated multimode fiber (M200L02S-A, Thorlabs), filtered through a 550/49 nm single band-pass filter (Semrock), and dispersed into spectra by a polychromator (Becker and Hickl). Individual photons were detected and recorded by a time-correlated single-photon counting (TCSPC) module (Becker and Hickl) at a frequency of 50 Hz. The shape, location, and amplitude of the TCSPC-derived fluorescence spectrum were used to confirm in vivo GCaMP expression.

A TDT RZ5P fiber photometry system and Synapse software were also used in this study. Briefly, light from the 470 nm, 17.2 mW (Min) fiber-coupled LED (Thorlabs) and light from the 405 nm, 19.3 mW (Min) fiber-coupled LED (Thorlabs) was directed into a fluorescence mini cube with six ports and a built-in detector head (Doric Lenses), with spectral bandwidths of 405 and 470 nm. A 405 nm light was modulated at 217 Hz, while 470 nm light was modulated at 330 Hz. Power output was maintained at 20 mA with a DC offset of 3 mA for both wavelengths. The light was then directed through a low-autofluorescence mono fiberoptic patch cord with a 400 µm core (Precision Fiber Products). This fiber was connected to the mono fiberoptic cannulas that were implanted into the region of interest. The power output at the fiber tip was 25–30 µW. Fluorescent emission from the tissue was collected through the same fiber and was detected using a femtowatt photoreceiver. Signal acquisition was 1 kHz and low-pass filtered at 6 Hz. The 405 nm excitation channel served as an isosbestic, calcium-independent control wavelength for GCaMP, allowing for bleaching and movement artifact corrections when directly fit to the calcium-dependent 470 nm channel. MATLAB scripts from TDT (https://www.tdt.com/support/matlab-sdk/) were used to fit the 405 nm signal to the 470 nm signal using linear regression. Change in GCaMP-mediated signal was calculated as

1 $$\triangle {\rm{F}}/{\rm{F}}=\frac{{{\rm{change}}}\; {\rm{in}}\;470\,{\rm{nm}}\; {\rm{induced}}\; {\rm{signal}}/{\rm{change}}\; {\rm{in}}\;405\,{\rm{nm}}\; {\rm{induced}}\; {\rm{signal}}}{{{\rm{change}}\; {\rm{in}}\;405\,{\rm{nm}}\; {\rm{induced}}\; {\rm{signal}}}}$$

Time-locked *Z* scores were then calculated from GCaMP-mediate signal as

2 $${{Z}}=\frac{{\rm{instantaneous}}\;\frac{\triangle {\rm{F}}}{{\rm{F}}}/{\rm{mean}}\;\frac{\triangle {\rm{F}}}{{\rm{F}}}\;({\rm{from}}-\!5\;{\rm{to}}\;-\!3\,{\rm{s}})}{{\rm{standard}}\; {\rm{deviation}}\; {\rm{of}}\;\frac{\triangle {\rm{F}}}{{\rm{F}}}\;({\rm{from}}-\!5\;{\rm{to}}\;-\!3\,{\rm{s}})}$$

### Methods for frequency and Max peak detection

Transient frequency and maximum peak amplitude were calculated using a custom written MATLAB code incorporating open-source code^59^ (https://github.com/katemartian/Photometry_data_processing). The pipeline utilized an adaptive iteratively reweighted Penalized Least Square (airPLS) approach^60^ to correct for baseline noise or signal drift, and allow for uniform detection of transients and comparison between mice. The signal was then standardized using a *Z* score transformation and fit to the reference signal (405 nm, GCaMP only). Data points with a *Z* score ≥ 2.91 are considered statistical outliers from the baseline (i.e., transient activity above baseline)^61^. A single transient was measured from when a data point ≥2.91 *Z* scores was detected until a data point <2.91 *Z* scores was detected. Maximum peak amplitude was calculated as the maximum *Z* score detecting during the transient. Transient frequency was calculated as the number of transients per second (Hz).

### Brain clearing

SHIELD (Stabilization under Harsh conditions via Intramolecular Epoxide Linkages to prevent Degradation)^62^. Mouse brain tissue was prepared according to the LifeCanvas SHIELD protocol^62,63^. Mice were anesthetized with isoflurane and transcardially perfused with ice-cold PBS followed by 20 mL of ice-cold SHIELD perfusion solution. Brains were then dissected out and shaken in the remaining 20 mL of SHIELD perfusion solution at 4 °C for 48 h. Brains were switched to fresh 20 mL of SHIELD-OFF solution and shaken for 24 h at 4 °C. Brains were then shaken in SHIELD-ON for 24 h at 37 °C.

SHIELD perfusion solution was prepared fresh before all perfusions. For each mouse, a total of 40 mL was made by mixing 5 mL DI water, 10 mL SHIELD-buffer, 5 mL of 32% PFA, and 20 mL SHIELD-Epoxy (added in 10 mL increments). Solutions were kept on ice and vortexed after each new reagent was added.

SHIELD-OFF solution was prepared fresh before use. For each mouse, a total of 20 mL was made by mixing 5 mL DI water, 50 mL SHIELD-buffer, and 10 mL SHIELD-Epoxy.

#### Active clearing

Brains were cleared using the SmartClear II Pro (LifeCanvas) following the SmartClear II Pro User’s Manual^64^, in beginner mode (limit = 85 V, current = 1500 mA, buffer A temp. = 42 °C, buffer B temp. = 30–40 °C). This method uses stochastic electrotransport to remove electromobile molecules, such as phospholipids, from the sample^62,65^. A maximum of four brains were cleared until evenly transparent throughout (4–6 days).

#### Passive clearing

Brains were transferred to passive SDS clearing solution and shaken at 37 °C until fully transparent (3–4 weeks). The passive clearing solution was changed once per week. The passive SDS clearing solution was prepared as a 2 L batch prior to clearing. A total of 173 g of SDS (300 mM), 1.24 g of sodium borate (10 mM), and 25.2 g sodium sulfate (100 mM) were mixed in Milli-Q filtered water (final volume = 2 L, pH = 9).

#### Mounting for imaging

After clearing, brains were shaken in PBS at 37 °C for 12 h. The samples were then shaken in EasyIndex (LifeCanvas) refractive index (RI) matching solution overnight at 37 °C. Custom sample holders were rinsed with DI H_2_O, followed by 70% ethanol, and allowed to evaporatively dry. The sample holder (LifeCanvas) was filled with RI-matched agarose (Sigma-Aldrich). The brains were then placed in a sample holder, which was then quickly transferred to a 4 °C cold room to congeal for ~15 min. Brains were shaken in 50 mL conical tubes with 25 mL of EasyIndex for ~4 h at 37 °C, and then allowed to equilibrate in the imaging.

### Light sheet microscopy

#### Imaging

Whole-brain light sheet images were captured with a selective plane illumination microscope (SmartSpim, LifeCanvas Technologies), incorporating axially swept light sheet generation^66,67^ and a 3.6× objective (NA = 0.2). Samples were illuminated with up to three excitation wavelengths: 488, 561, and 642 nm. Respective emission detection filters were 525/50, 600/52, and 680/42. Laser power was set to 30–55% for each channel, and the step size was set to 2 µm (Nyquist sampling for the ~4 µm axial point spread function for the 3.6× objective). Following acquisition, images were transferred to a Dell Precision 7920 Tower with (2) Intel Xeon Gold 6134 CPU at 3.2 GHz and 384 GB of RAM running Windows 10 Pro for Workstations. Images were stitched to generate composite TIFF images by using a modified version of Terastitcher^68^. Stitched TIFF images were converted to Imaris files using Imaris File Converter 9.2.1 for visualization, using Imaris 9.5.1.

### Brain registration and cell quantification

The distribution of virally transduced neurons was registered to the Allen Coordinate Reference Framework^32^ and regional densities of labeled cells quantified, using NeUroGlancer Ground Truth (NUGGT)^69^ (Supplementary Fig. 4c), with custom modifications provided by LifeCanvas Technologies. A minimum of 200 cells per brain were trained in the NUGGT pipeline.

### Electrophysiology

We used whole-cell patch-clamp electrophysiological techniques^26,70^ to interrogate the circuit. Mice were deeply anesthetized using isoflurane, transcardially perfused with ice-cold sucrose-based artificial cerebrospinal fluid (ACSF; in mM: 194 sucrose, 20 NaCl, 4.4 KCl, 2 CaCl_2_, MgCl_2_, 1.2 NaH_2_PO_4_, 10 glucose, and 26 NaHCO_3_). Mice were decapitated, and the brains were removed and immediately placed in a holding chamber containing ice-cold sucrose ACSF. Acute coronal slices (300 µM) containing the insula were cut on a Leica vibratome. Slices were transferred to a holding chamber and incubated for 1 h at 28 °C in normal oxygenated ACSF (in mM: 124 NaCl, 4.4 KCl, 2.5 CaCl_2_, 1.3 MgSO_4_, 1 NaH_2_PO_4_, 10 glucose, 26 and NaHCO_3_). In the recording chamber, slices were continuously perfused with oxygenated and heated (28 °C) ACSF at a rate of 1–2 mL/min. Whole-cell voltage-clamp recordings were performed using electrodes (2.5–5.0 MΩ) filled with internal ACSF (in mM: 135 K+-gluconate, 5 NaCl, 10 HEPES, 0.6 EGTA, and 4 Na_2_GTP (pH 7.2–7.4, osmolarity 290–295)). Fluorescently labeled (via AAVrg.DIO.tdTomato injection into the BNST and AAV1.Cre injection into the motor cortex) insula cells were held at −70 mV throughout the recording, and all recordings were done in the presence of picrotoxin to block GABA signaling (25 µM; MilliporeSigma; St. Louis, MO). Postsynaptic parameters were monitored continuously during the experiments, and cells were excluded if the access resistance (*R*_a_) changed by >20% in either direction.

### RNA fluorescent in situ hybridization

Fluorescent in situ hybridization assays were performed using RNA-Scope Fluorescent Multiplex Reagent Kit (Advanced Cell Diagnostics) to visualize RNA transcripts in BNST coronal sections^22^. Isoflurane was used to anesthetize mice immediately prior to brain extraction. Brains were then quickly submerged in oxygenated (95% O_2_ and 5% CO_2_), ice-cold ACSF. ACSF contained 124 mM NaCl, 4.4 mM KCl, 2.5 mM CaCl_2_, 1.3 mM MgSO_4_, 1 mM NaH_2_PO_4_, 10 mM glucose, and 26 mM NaHCO_3_ in Milli-Q filtered H_2_O. Brains were immediately flash-frozen in Optimal Cutting Temperature (OCT) Solution (VWR), using Super Friendly Freeze-It Spray (Fisher Scientific). OCT-embedded brains were kept at −80 °C until they were sliced on a cryostat (Leica). A total of 16 µm slices were stuck to charged slides (Denville Scientific), frozen on dry ice, and stored at −80 °C until staining. Fixation, dehydration, hybridization, and staining protocols for fresh-frozen tissue were performed, according to ACD’s online specifications. *Z*-stack BNST images were obtained with a 63×/1.4 NA oil lens on a Zeiss 710 scanning confocal microscope. Three images were taken to visualize the medial, lateral, and dorsal BNST (Bregma –0.14). The negative control probe, DapB, was used to determine the parameters for brightness and contrast for experimental images. Images were processed as max intensity projections with Fiji software (NIH)^71^. Counts from the medial, lateral, and dorsal images were combined for each animal. Cells were identified using DAPI-labeled nuclei, and transcripts were identified as individual dots within a cell. A blinded reviewer classified cells as positive (at least one dot in the cell) or negative (zero dots in the cell). Probes used include Mm-Crh-C1 and Mm-Fos-C2. Negative control images were used to determine thresholding parameters that excluded nonspecific fluorescence.

### Statistical analysis

Analyses were performed with R (version 3.6.1), Python (version 3.6.6), GraphPad Prism (GraphPad Software, version 8), or MATLAB (Mathworks; version 2019a). Animal numbers were selected to establish sufficient statistical power, while using ethical guidelines for minimizing subject numbers and based on previous publications’ ability to reliably measure variables from experiments with similar design. When manual scoring was performed, the scorer was blinded to the treatment. For in situ hybridization, images like that shown in Fig. 3b were collected for each animal. When automated behavior tracking was performed, a custom R script was used to determine bout onset in an unbiased manner. Fiberoptic implants and viral placements were histologically verified, and animals were removed if either did not work. The number of mice in each experiment and the specific statistical tests are identified in the corresponding figure legends. Statistical significance was determined if *P* ≤ 0.05.

### Reporting summary

Further information on research design is available in the Nature Research Reporting Summary linked to this article.

## Supplementary information

Supplementary Information

Description of Additional Supplementary Files

Supplementary Movie 1 BNST AAVrg-tdTomato

Supplementary Movie 2 BNST AAVrg-tdTomato Cell Count

Supplementary Movie 3 BNST AAVrg-tdTomato Density

Supplementary Movie 4 AAV1-Cre Insula in Ai14mouse

Supplementary Movie 5 TRIO Insula→BNST

Supplementary Movie 6 TRIO Insula→BNST Cell Count

Supplementary Movie 7 TRIO Insula→BNST Density

Supplementary Movie 8 Insula AAVrg-tdTomato

Supplementary Movie 9 Insula AAVrg-tdTomato Cell Count

Supplementary Movie 10 Insula AAVrg-tdTomato Density

Supplementary Movie 11 Motor AAV1-Cre Ai14mouse

Supplementary Movie 12 BNST AAVrg-GFP, Motor AAV1-Cre Ai14mouse

Reporting Summary

## Data Availability

The data that support the findings of this study are available from the corresponding authors upon reasonable request. Source data are provided with this paper.

## Source data

Source Data

## Code availability

The code that supports the findings of this study is available from the corresponding author upon reasonable request.

## References

[1] Carroll, L. in *Encyclopedia of Behavioral Medicine* (eds. Turner, J. R. & Gellman, M. D.) (Springer, 2013).

[2] Wood, S. K. & Bhatnagar, S. Resilience to the effects of social stress: evidence from clinical and preclinical studies on the role of coping strategies. *Neurobiol. Stress*. 10.1016/j.ynstr.2014.11.002 (2015). 10.1016/j.ynstr.2014.11.002 PMC4286805 25580450

[3] McEwen, B. S. et al. Mechanisms of stress in the brain. *Nat. Neurosci*. 10.1038/nn.4086 (2015). 10.1038/nn.4086 PMC4933289 26404710

[4] Kim, J. S., Han, S. Y. & Iremonger, K. J. Stress experience and hormone feedback tune distinct components of hypothalamic CRH neuron activity. *Nat. Commun*. 10.1038/s41467-019-13639-8 (2019). 10.1038/s41467-019-13639-8 PMC6911111 31836701

[5] Daviu, N. et al. Paraventricular nucleus CRH neurons encode stress controllability and regulate defensive behavior selection. *Nat. Neurosci.*10.1038/s41593-020-0591-0 (2020). 10.1038/s41593-020-0591-0 32066984

[6] Gogolla, N. The insular cortex. *Curr. Biol.*10.1016/j.cub.2017.05.010 (2017). 10.1016/j.cub.2017.05.010 28633023

[7] Johnson, S. B. et al. Prefrontal–bed nucleus circuit modulation of a passive coping response set. *J. Neurosci*. 10.1523/JNEUROSCI.1421-18.2018 (2019). 10.1523/JNEUROSCI.1421-18.2018 PMC6381256 30573644

[8] Gehrlach, D. A. et al. Aversive state processing in the posterior insular cortex. *Nat. Neurosci.*10.1038/s41593-019-0469-1 (2019). 10.1038/s41593-019-0469-1 31455886

[9] Giardino, W. J. et al. Parallel circuits from the bed nuclei of stria terminalis to the lateral hypothalamus drive opposing emotional states. *Nat. Neurosci*. 10.1038/s41593-018-0198-x (2018). 10.1038/s41593-018-0198-x PMC6095688 30038273

[10] Van Praag, H., Christie, B. R., Sejnowski, T. J. & Gage, F. H. Running enhances neurogenesis, learning, and long-term potentiation in mice. *Proc. Natl. Acad. Sci. USA*. 10.1073/pnas.96.23.13427 (1999). 10.1073/pnas.96.23.13427 PMC23964 10557337

[11] Patel, S., Roelke, C. T., Rademacher, D. J. & Hillard, C. J. Inhibition of restraint stress-induced neural and behavioural activation by endogenous cannabinoid signalling. *Eur. J. Neurosci.*10.1111/j.1460-9568.2005.03916.x (2005). 10.1111/j.1460-9568.2005.03916.x 15787710

[12] Miles, O. W. & Maren, S. Role of the bed nucleus of the stria terminalis in PTSD: insights from preclinical models. *Fron. Behav. Neurosci.*10.3389/fnbeh.2019.00068 (2019). 10.3389/fnbeh.2019.00068 PMC6461014 31024271

[13] Grissom, N., Kerr, W. & Bhatnagar, S. Struggling behavior during restraint is regulated by stress experience. *Behav. Brain Res*. 10.1016/j.bbr.2008.03.030 (2008). 10.1016/j.bbr.2008.03.030 PMC2477735 18466984

[14] Mathis, A. et al. DeepLabCut: markerless pose estimation of user-defined body parts with deep learning. *Nat. Neurosci*. 10.1038/s41593-018-0209-y (2018). 10.1038/s41593-018-0209-y 30127430

[15] Nath, T. et al. Using DeepLabCut for 3D markerless pose estimation across species and behaviors. *Nat. Protoc*. 10.1038/s41596-019-0176-0 (2019). 10.1038/s41596-019-0176-0 31227823

[16] Moreira, F. A., Grieb, M. & Lutz, B. Central side-effects of therapies based on CB1 cannabinoid receptor agonists and antagonists: focus on anxiety and depression. *Best Pract. Res. Clin. Endocrinolo. Metabol.*10.1016/j.beem.2008.09.003 (2009). 10.1016/j.beem.2008.09.003 19285266

[17] Blasio, A. et al. Rimonabant precipitates anxiety in rats withdrawn from palatable food: role of the central Amygdala. *Neuropsychopharmacology*. 10.1038/npp.2013.153 (2013). 10.1038/npp.2013.153 PMC3799070 23793355

[18] Gamble-George, J. C. *et al*. Dissociable effects of CB1 receptor blockade on anxiety-like and consummatory behaviors in the novelty-induced hypophagia test in mice. *Psychopharmacology*. 10.1007/s00213-013-3042-8 (2013). 10.1007/s00213-013-3042-8 PMC3707973 23483200

[19] Marcinkiewcz CA (2016). Serotonin engages an anxiety and fear-promoting circuit in the extended amygdala. Nature.

[20] Olive, M. F., Koenig, H. N., Nannini, M. A. & Hodge, C. W. Elevated extracellular CRF levels in the bed nucleus of the stria terminalis during ethanol withdrawal and reduction by subsequent ethanol intake. *Pharmacol. Biochem. Behav*. 10.1016/S0091-3057(01)00748-1 (2002). 10.1016/s0091-3057(01)00748-1 PMC11583329 11900791

[21] Huang, M. M. et al. Corticotropin-releasing factor (CRF) sensitization of ethanol withdrawal-induced anxiety-like behavior is brain site specific and mediated by CRF-1 receptors: relation to stress-induced sensitization. *J. Pharmacol. Exp. Ther.*10.1124/jpet.109.159186 (2010). 10.1124/jpet.109.159186 PMC2802475 19843974

[22] Fetterly, T. L. et al. α 2A -adrenergic receptor activation decreases parabrachial nucleus excitatory drive onto BNST CRF neurons and reduces their activity in vivo. *J. Neurosci.*10.1523/JNEUROSCI.1035-18.2018 (2019). 10.1523/JNEUROSCI.1035-18.2018 PMC6335747 30478032

[23] Snyder, A. E., Salimando, G. J., Winder, D. G. & Silberman, Y. Chronic intermittent ethanol and acute stress similarly modulate BNST CRF neuron activity via noradrenergic signaling. *Alcohol. Clin. Exp. Res*. 10.1111/acer.14118 (2019). 10.1111/acer.14118 PMC6677590 31141179

[24] Silberman Y, Matthews RT, Winder DG (2013). A corticotropin releasing factor pathway for ethanol regulation of the ventral tegmental area in the bed nucleus of the stria terminalis. J. Neurosci..

[25] Marvin, J. S. et al. Stability, affinity, and chromatic variants of the glutamate sensor iGluSnFR. *Nat. Methods*. 10.1038/s41592-018-0171-3 (2018). 10.1038/s41592-018-0171-3 PMC6394230 30377363

[26] Centanni, S. W. S. W. et al. Endocannabinoid control of the insular-bed nucleus of the stria terminalis circuit regulates negative affective behavior associated with alcohol abstinence. *Neuropsychopharmacology* **44**, 526–537 (2019). 10.1038/s41386-018-0257-8 PMC6333805 30390064

[27] Lebow, M. A. & Chen, A. Overshadowed by the amygdala: the bed nucleus of the stria terminalis emerges as key to psychiatric disorders. *Mol. Psychiatry*. 10.1038/mp.2016.1 (2016). 10.1038/mp.2016.1 PMC4804181 26878891

[28] Marvin, J. S. et al. A genetically encoded fluorescent sensor for in vivo imaging of GABA. *Nat. Methods*. 10.1038/s41592-019-0471-2 (2019). 10.1038/s41592-019-0471-2 31308547

[29] Patel, S., Kingsley, P. J., MacKie, K., Marnett, L. J. & Winder, D. G. Repeated homotypic stress elevates 2-arachidonoylglycerol levels and enhances short-term endocannabinoid signaling at inhibitory synapses in basolateral amygdala. *Neuropsychopharmacology*. 10.1038/npp.2009.101 (2009). 10.1038/npp.2009.101 PMC2881681 19675536

[30] Morgan, A. et al. Cyclooxygenase-2 inhibition reduces anxiety-like behavior and normalizes enhanced amygdala glutamatergic transmission following chronic oral corticosterone treatment. *Neurobiol. Stress*10.1016/j.ynstr.2019.100190 (2019). 10.1016/j.ynstr.2019.100190 PMC6710559 31467944

[31] Schwarz LA (2015). Viral-genetic tracing of the input-output organization of a central noradrenaline circuit. Nature.

[32] Oh, S. W. et al. A mesoscale connectome of the mouse brain. *Nature*. 10.1038/nature13186 (2014). 10.1038/nature13186 PMC5102064 24695228

[33] Petreanu, L., Huber, D., Sobczyk, A. & Svoboda, K. Channelrhodopsin-2-assisted circuit mapping of long-range callosal projections. *Nat. Neurosci.*10.1038/nn1891 (2007). 10.1038/nn1891 17435752

[34] Davis, M., Walker, D. L., Miles, L. & Grillon, C. Phasic vs sustained fear in rats and humans: role of the extended amygdala in fear vs anxiety. *Neuropsychopharmacology* **35**, 105–135 (2010). 10.1038/npp.2009.109 PMC2795099 19693004

[35] Silberman, Y. & Winder, D. G. Emerging role for corticotropin releasing factor signaling in the bed nucleus of the stria terminalis at the intersection of stress and reward. *Front. Psychiatry*. 10.3389/fpsyt.2013.00042 (2013). 10.3389/fpsyt.2013.00042 PMC3665954 23755023

[36] Di Bonaventura, M. V. M. et al. Role of bed nucleus of the stria terminalis Corticotrophin-Releasing factor receptors in frustration stress-induced binge-like palatable food consumption in female rats with a history of food restriction. *J. Neurosci*. 10.1523/JNEUROSCI.1854-14.2014 (2014). 10.1523/JNEUROSCI.1854-14.2014 PMC4138341 25143612

[37] Sahuque, L. L. et al. Anxiogenic and aversive effects of corticotropin-releasing factor (CRF) in the bed nucleus of the stria terminalis in the rat: role of CRF receptor subtypes. *Psychopharmacology*. 10.1007/s00213-006-0362-y (2006). 10.1007/s00213-006-0362-y PMC1473306 16568282

[38] Kayyal, H. et al. Activity of insula to basolateral amygdala projecting neurons is necessary and sufficient for taste valence representation. *J. Neurosci*. 10.1523/JNEUROSCI.0752-19.2019 (2019). 10.1523/JNEUROSCI.0752-19.2019 PMC6867822 31597726

[39] Flook, E. A. et al. BNST-insula structural connectivity in humans. *Neuroimage*. 10.1016/j.neuroimage.2020.116555 (2020). 10.1016/j.neuroimage.2020.116555 PMC7089680 31954845

[40] Gerfen, C. R. & Clavier, R. M. Neural inputs to the prefrontal agranular insular cortex in the rat: Horseradish peroxidase study. *Brain Res. Bull*. 10.1016/S0361-9230(79)80012-X (1979). 10.1016/s0361-9230(79)80012-x 90546

[41] Gehrlach, D. A. et al. A whole-brain connectivity map of mouse insular cortex. *Elife*. 10.7554/ELIFE.55585 (2020). 10.7554/eLife.55585 PMC7538160 32940600

[42] Reynolds, S. M. & Zahm, D. S. Specificity in the projections of prefrontal and insular cortex to ventral striatopallidum and the extended amygdala. *J. Neurosci*. 10.1523/JNEUROSCI.3432-05.2005 (2005). 10.1523/JNEUROSCI.3432-05.2005 PMC6726011 16354934

[43] Zingg B (2017). AAV-mediated anterograde transsynaptic tagging: mapping corticocollicular input-defined neural pathways for defense behaviors. Neuron.

[44] Zingg, B., Peng, B., Huang, J., Tao, H. W. & Zhang, L. I. Synaptic specificity and application of anterograde transsynaptic AAV for probing neural circuitry. *J. Neurosci*. 10.1523/JNEUROSCI.2158-19.2020 (2020). 10.1523/JNEUROSCI.2158-19.2020 PMC7159884 32198185

[45] Zschucke, E., Renneberg, B., Dimeo, F., Wüstenberg, T. & Ströhle, A. The stress-buffering effect of acute exercise: Evidence for HPA axis negative feedback. *Psychoneuroendocrinology*. 10.1016/j.psyneuen.2014.10.019 (2015). 10.1016/j.psyneuen.2014.10.019 25462913

[46] Chen, C. et al. The exercise-glucocorticoid paradox: how exercise is beneficial to cognition, mood, and the brain while increasing glucocorticoid levels. *Front. Neuroendocrinol.*10.1016/j.yfrne.2016.12.001 (2017). 10.1016/j.yfrne.2016.12.001 27956050

[47] Moylan, S. et al. Exercising the worry away: how inflammation, oxidative and nitrogen stress mediates the beneficial effect of physical activity on anxiety disorder symptoms and behaviours. *Neurosci. Biobehav. Rev.*10.1016/j.neubiorev.2013.02.003 (2013). 10.1016/j.neubiorev.2013.02.003 23415701

[48] Eyre, H. & Baune, B. T. Neuroimmunological effects of physical exercise in depression. *Brain Behav. Immunity*. 10.1016/j.bbi.2011.09.015 (2012). 10.1016/j.bbi.2011.09.015 21986304

[49] Wolpert, D. M. & Miall, R. C. Forward models for physiological motor control. *Neural Netw.* **9**, 1265–1279 (1996). 10.1016/s0893-6080(96)00035-4 12662535

[50] Blakemore, S. J., Wolpert, D. & Frith, C. Why can’t you tickle yourself? *NeuroReport*. 10.1097/00001756-200008030-00002 (2000). 10.1097/00001756-200008030-00002 10943682

[51] Whitford, T. J. et al. Neurophysiological evidence of efference copies to inner speech. *Elife*. 10.7554/eLife.28197 (2017). 10.7554/eLife.28197 PMC5714499 29199947

[52] Blakemore, S. J., Smith, J., Steel, R., Johnstone, E. C. & Frith, C. D. The perception of self-produced sensory stimuli in patients with auditory hallucinations and passivity experiences: evidence for a breakdown in self-monitoring. *Psychol. Med*. 10.1017/S0033291799002676 (2000). 10.1017/s0033291799002676 12027049

[53] Taniguchi H (2011). A resource of Cre driver lines for genetic targeting of GABAergic. Neuron.

[54] Madisen, L. et al. A robust and high-throughput Cre reporting and characterization system for the whole mouse brain. *Nat. Neurosci*. 10.1038/nn.2467 (2010). 10.1038/nn.2467 PMC2840225 20023653

[55] R Core Team. *R: A Language and Environment for Statistical Computing* (R Foundation for Statistical Computing, 2019).

[56] Wickham, H. et al. Welcome to the Tidyverse. *J. Open Source Softw*. 10.21105/joss.01686 (2019).

[57] Kolde, R. pheatmap: pretty heatmaps. http://CRAN.R-project.org/package=pheatmap (2019).

[58] Wickham, H. *ggplot2: Elegant Graphics for Data Analysis* (Springer, 2016).

[59] Martianova, E., Aronson, S. & Proulx, C. D. Multi-fiber photometry to record neural activity in freely-moving animals. *J. Vis. Exp*. 10.3791/60278 (2019). 10.3791/60278 31680685

[60] Zhang, Z. M., Chen, S. & Liang, Y. Z. Baseline correction using adaptive iteratively reweighted penalized least squares. *Analyst*. 10.1039/b922045c (2010). 10.1039/b922045c 20419267

[61] Calipari, E. S. et al. In vivo imaging identifies temporal signature of D1 and D2 medium spiny neurons in cocaine reward. *Proc. Natl. Acad. Sci. USA*. 10.1073/pnas.1521238113 (2016). 10.1073/pnas.1521238113 PMC4791010 26831103

[62] Park, Y. G. et al. Protection of tissue physicochemical properties using polyfunctional crosslinkers. *Nat. Biotechnol*. 10.1038/nbt.4281 (2019). 10.1038/nbt.4281 PMC6579717 30556815

[63] LifeCanvas. SHIELD protocol with LifeCanvas devices. https://lifecanvastech.com/wp-content/uploads/2019 (2019).

[64] LifeCanvas. SmartClear II pro user’s manual. https://lifecanvastech.com/wp-content/uploads/2019 (2019).

[65] Kim, S. Y. et al. Stochastic electrotransport selectively enhances the transport of highly electromobile molecules. *Proc. Natl Acad. Sci. USA*. 10.1073/pnas.1510133112 (2015). 10.1073/pnas.1510133112 PMC4655572 26578787

[66] Hedde, P. N. & Gratton, E. Selective plane illumination microscopy with a light sheet of uniform thickness formed by an electrically tunable lens. *Microsc. Res. Tech*. **81**, 924–928 (2018). 10.1002/jemt.22707 PMC5479743 27338568

[67] Dean, K. M., Roudot, P., Welf, E. S., Danuser, G. & Fiolka, R. Deconvolution-free subcellular imaging with axially swept light sheet microscopy. *Biophys. J.*10.1016/j.bpj.2015.05.013 (2015). 10.1016/j.bpj.2015.05.013 PMC4472079 26083920

[68] Bria A, Bernaschi M, Guarrasi M, Iannello G (2019). Exploiting multi-level parallelism for stitching very large microscopy images. Front. Neuroinform..

[69] Swaney, J. et al. Scalable image processing techniques for quantitative analysis of volumetric biological images from light-sheet microscopy. Preprint at *bioRxiv*. 10.1101/576595 (2019).

[70] Harris, N. A. et al. Dorsal BNST α2a-adrenergic receptors produce HCN-dependent excitatory actions that initiate anxiogenic behaviors. *J. Neurosci*. 10.1523/JNEUROSCI.0963-18.2018 (2018). 10.1523/JNEUROSCI.0963-18.2018 PMC6191524 30150361

[71] Schindelin, J. et al. Fiji: An open-source platform for biological-image analysis. *Nat. Methods*. 10.1038/nmeth.2019 (2012). 10.1038/nmeth.2019 PMC3855844 22743772

