# Supplementary material for: Delineation of an insula-BNST circuit engaged by struggling behavior that regulates avoidance in mice
Source: Nat Commun. 2021 Jun 11;12:3561. doi: 10.1038/s41467-021-23674-z (PMC8196075; doi:10.1038/s41467-021-23674-z)
Supplement: Supplementary file 2 — Description of Additional Supplementary Files [file 41467_2021_23674_MOESM2_ESM.docx]

**Description of Additional Supplementary Files**

File Name: Supplementary Video 1 BNST AAVrg-tdTomato

Description: Example video of a mouse brain with a unilateral injection of AAVrg-tdTomato into the BNST.

File Name: Supplementary Video 2 BNST AAVrg-tdTomato Cell Count

Description: Heatmap of the average regional raw cell count from mouse brains after unilateral injection of AAVrg-tdTomato into the BNST. Coronal view moving from rostral to caudal.

File Name: Supplementary Video 3 BNST AAVrg-tdTomato

Description: Density Heatmap of the average regional cell density from mouse brains after unilateral injection of AAVrg-tdTomato into the BNST. Coronal view moving from rostral to caudal.

File Name: Supplementary Video 4 AAV1-Cre Insula in Ai14mouse

Description: Example video of an Ai14 mouse brain with a unilateral injection of AAV1-Cre into the insula.

File Name: Supplementary Video 5 TRIO Insula→BNST

Description: Example video of a TRIO (for the insula→BNST pathway) mouse brain.

File Name: Supplementary Video 6 TRIO Insula→BNST Cell Count

Description: Heatmap of the average regional raw cell count from TRIO (for the insula→BNST pathway) mouse brains. Coronal view moving from rostral to caudal.

File Name: Supplementary Video 7 TRIO Insula→BNST Density

Description: Heatmap of the average regional cell density from TRIO (for the insula→BNST pathway) mouse brains. Coronal view moving from rostral to caudal.

File Name: Supplementary Video 8 Insula AAVrg-tdTomato

Description: Example video of a mouse brain with a unilateral injection of AAVrg-tdTomato into the insula.

File Name: Supplementary Video 9 Insula AAVrg-tdTomato Cell Count

Description: Heatmap of the average regional raw cell count from mouse brains after unilateral injection of AAVrg-tdTomato into the insula. Coronal view moving from rostral to caudal.

File Name: Supplementary Video 10 Insula AAVrg-tdTomato Density

Description: Heatmap of the average regional cell density from mouse brains after unilateral injection of AAVrg-tdTomato into the insula. Coronal view moving from rostral to caudal.

File Name: Supplementary Video 11 Motor AAV1-Cre Ai14mouse

Description: Example video of an Ai14 mouse brain with a unilateral injection of AAV1-Cre into the motor cortex.

File Name: Supplementary Video 12 BNST AAVrg-GFP, Motor AAV1-Cre Ai14mouse

Description: Example video of an Ai14 mouse brain with a unilateral injection of AAVrg-eGFP into the BNST and an ipsilateral injection of AA1-Cre in to the motor cortex.
