# Supplementary material for: Delineation of an insula-BNST circuit engaged by struggling behavior that regulates avoidance in mice
Source: Nat Commun. 2021 Jun 11;12:3561. doi: 10.1038/s41467-021-23674-z (PMC8196075; doi:10.1038/s41467-021-23674-z)
Supplement: Supplementary file 15 — Reporting Summary [file 41467_2021_23674_MOESM15_ESM.pdf]

nature research

Corresponding author(s): Danny Winder

Last updated by author(s): Apr 30, 2021

Reporting Summary

Nature Research wishes to improve the reproducibility of the work that we publish. This form provides structure for consistency and transparency in reporting. For further information on Nature Research policies, see our Editorial Policies and the Editorial Policy Checklist.

Statistics

For all statistical analyses, confirm that the following items are present in the figure legend, table legend, main text, or Methods section.

n/a	Confirmed	
☐	☒	The exact sample size (*n*) for each experimental group/condition, given as a discrete number and unit of measurement
☐	☒	A statement on whether measurements were taken from distinct samples or whether the same sample was measured repeatedly
☐	☒	The statistical test(s) used AND whether they are one- or two-sided<br>*Only common tests should be described solely by name; describe more complex techniques in the Methods section.*
☐	☒	A description of all covariates tested
☐	☒	A description of any assumptions or corrections, such as tests of normality and adjustment for multiple comparisons
☐	☒	A full description of the statistical parameters including central tendency (e.g. means) or other basic estimates (e.g. regression coefficient) AND variation (e.g. standard deviation) or associated estimates of uncertainty (e.g. confidence intervals)
☐	☒	For null hypothesis testing, the test statistic (e.g. *F*, *t*, *r*) with confidence intervals, effect sizes, degrees of freedom and *P* value noted<br>*Give P values as exact values whenever suitable.*
☐	☒	For Bayesian analysis, information on the choice of priors and Markov chain Monte Carlo settings
☐	☒	For hierarchical and complex designs, identification of the appropriate level for tests and full reporting of outcomes
☐	☒	Estimates of effect sizes (e.g. Cohen's *d*, Pearson's *r*), indicating how they were calculated

Our web collection on statistics for biologists contains articles on many of the points above.

Software and code

Policy information about availability of computer code

Data collection	Data collection used the following software. LifeCanvas Technologies SmartSpim software 3.5 was used for light sheet acquisition. Images were stitched to generate composite TIFF images by using the open-source software Terastitcher. Stitched TIFF images were converted to Imaris files using Imaris File Converter 9.2.1for visualization using Imaris 9.5.1.<br>Initial behavioral data were collected with Anymaze 5.3 software.<br>Photometry data was collected using Becker & Hickle SPCM Software 9.83 or TDT Synapse Software (Build: 92-40764p) software, as noted in the manuscript.
Data analysis	ImageJ/FIJI 2.0 was used for histological analysis.<br><br>Initial behavioral scoring was done using the open-source software Anvil 6.0.<br><br>Automated video scoring was performed using DeepLabCut (DLC) 2.1 to track points across time and was based on the methods used for manual scoring, as described in the manuscript. DLC is open source and readily available.<br><br>Light sheet visualization was completed using the commercial software Imaris (9.5.1).<br><br>We used the open-source R statistical software with the tidyverse package to convert X/Y position into fiber and tail speed. The R code used to perform this calculation will be made available to editors, reviewers, and other interested parties (including readers' requests if the manuscript is published) upon request.<br><br>Light-dark behavior analyzed with Prism 8.

We used R with the pheatmap package to create the time-locked photometry heatmaps.

Figures were generated in R with the ggplot2 package. All code will be made available on request.
The distribution of virally transduced neurons was registered to the Allen Coordinate Reference Framework and regional densities of labeled cells quantified using NeUroGlancer Ground Truth (NUGGT; https://github.com/chunglabmit/nuggt), with custom modifications provided by LifeCanvas Technologies. NUGGT is readily available through the public code repository GitHub, and the custom version that we used is available through the commercial vendor, LifeCanvas Technologies.
MATLAB (2019a) scripts 473 from TDT (https://www.tdt.com/support/matlab-sdk/) were used to fit the 405 nm signal to the 470 nm signal using linear regression.

For manuscripts utilizing custom algorithms or software that are central to the research but not yet described in published literature, software must be made available to editors and reviewers. We strongly encourage code deposition in a community repository (e.g. GitHub). See the Nature Research guidelines for submitting code & software for further information.

Data

Policy information about availability of data

All manuscripts must include a data availability statement. This statement should provide the following information, where applicable:

- Accession codes, unique identifiers, or web links for publicly available datasets
- A list of figures that have associated raw data
- A description of any restrictions on data availability

The data that support the findings of this study are available from the corresponding author, DGW, upon reasonable request.

Field-specific reporting

Please select the one below that is the best fit for your research. If you are not sure, read the appropriate sections before making your selection.

☒ Life sciences ☐ Behavioural & social sciences ☐ Ecological, evolutionary & environmental sciences

For a reference copy of the document with all sections, see nature.com/documents/nr-reporting-summary-flat.pdf

Life sciences study design

All studies must disclose on these points even when the disclosure is negative.

Sample size	Sample size was determined by examining previous work in our lab and along with other similar studies. We used those effect sizes and the expected effect size in our work, to inform our sample numbers here.
Data exclusions	Animals were excluded from photometry experiments if viral fiber placement were not in the appropriate location as verified by histological sectioning. This was pre-established.
Replication	We used a variety of convergent techniques to bolster our confidence in the reproducibility of the findings in this work. For studies that a occurred across multiple days, we found consistent results across days.
Randomization	In experiments with different treatment groups, animals were randomly assigned a treatment group.
Blinding	Scorers were blinded when hand scoring was implemented. For non-hand scored analysis, blinding the scorer was not relevant because the scoring was performed autonomously.

Reporting for specific materials, systems and methods

We require information from authors about some types of materials, experimental systems and methods used in many studies. Here, indicate whether each material, system or method listed is relevant to your study. If you are not sure if a list item applies to your research, read the appropriate section before selecting a response.

Materials & experimental systems

n/a	Involved in the study	
☒	☐	Antibodies
☒	☐	Eukaryotic cell lines
☒	☐	Palaeontology and archaeology
☐	☒	Animals and other organisms
☒	☐	Human research participants
☒	☐	Clinical data
☒	☐	Dual use research of concern

Methods

n/a	Involved in the study	
☒	☐	ChIP-seq
☒	☐	Flow cytometry
☒	☐	MRI-based neuroimaging

Animals and other organisms

Policy information about studies involving animals; ARRIVE guidelines recommended for reporting animal research

Laboratory animals	Mice: C57BL/6J (The Jackson Laboratory, RRID:IMSR_JAX:000664); B6(Cg)-Crhtm1(cre)Zjh/J (The Jackson Laboratory, RRID:IMSR_JAX: 012704); Tg(Prkcd-glc-1/CFP,- cre)EH124Gsat (The Jackson Laboratory,RRID:MMRRC_011559- UCD); B6;129S6- Gt(ROSA)26Sortm14 (CAG-tdTomato)Hze/J (The Jackson Laboratory, RRID:IMSR_JAX: 007908) (male and female mice of at least 8 weeks in age)
Wild animals	No wild animals were used.
Field-collected samples	No field-collected samples were used.
Ethics oversight	The mouse work was performed under the study protocol M1800046-00 as approved by Vanderbilt's Institutional Animal Care and Use Committee.

Note that full information on the approval of the study protocol must also be provided in the manuscript.
