# Supplementary material for: Delineation of an insula-BNST circuit engaged by struggling behavior that regulates avoidance in mice
Source: Nat Commun. 2021 Jun 11;12:3561. doi: 10.1038/s41467-021-23674-z (PMC8196075; doi:10.1038/s41467-021-23674-z)
Supplement: Supplementary file 1 — Supplementary Information [file 41467_2021_23674_MOESM1_ESM.pdf]

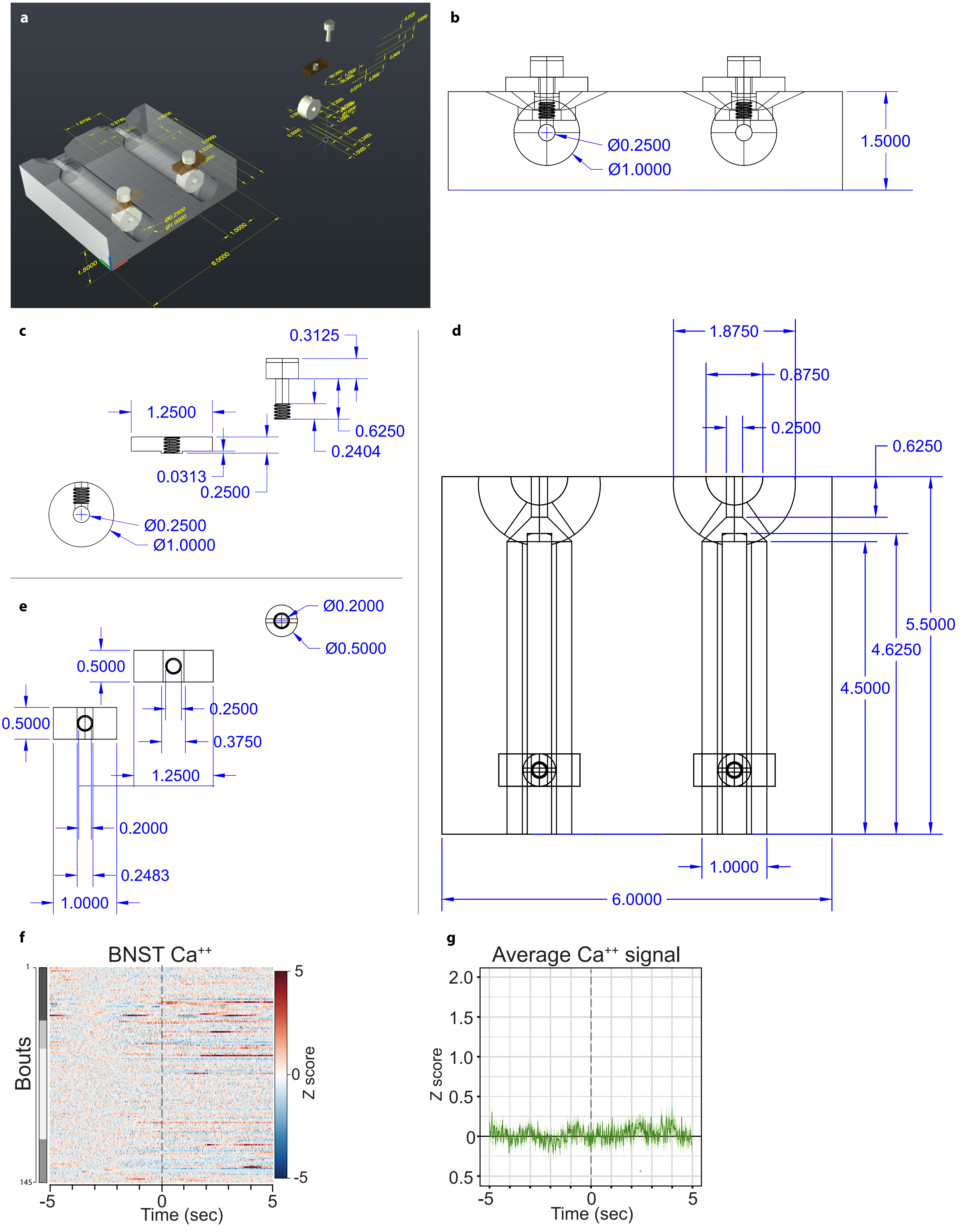

a
b
Ø0.2500
Ø1.0000
1.5000
c
0.3125
1.2500
0.6250
0.2404
0.0313
0.2500
Ø0.2500
Ø1.0000
d
1.8750
0.8750
0.2500
0.6250
5.5000
4.6250
4.5000
1.0000
6.0000
e
Ø0.2000
Ø0.5000
0.5000
0.5000
0.2500
0.3750
1.2500
0.2000
0.2483
1.0000
f
BNST Ca++
Bouts
1
145
Z score
5
0
-5
-5
0
5
Time (sec)
g
Average Ca++ signal
2.0
1.5
1.0
0.5
0
0.5
Z score
-5
0
5
Time (sec)

Supplementary Figure 1: RESTRAINT device design and initial BNST-GCaMP control signal.

a, Overview of computer-aided design (CAD) of RESTRAINT (**RE**cording **S**ignal **TR**ansients **A**ccessible **IN** a **T**ube) device for concurrent *in vivo* recordings. All indicated measurements are in inches. **b**, Front view of the CAD diagram of the RESTRAINT device. Black lines indicate edges of the device. Blue lines and text are measurements. **c**, Front view of the bung for the RESTRAINT. **d**, Top view of the RESTRAINT device. **e**, Top view of the bung for the RESTRAINT. **f**, Control heatmap for GCaMP signals in the BNST. Instead of GCaMP signal being time-locked to bout onset, the GCaMP signals were offset from bouts by +10 seconds. **g**, Average of the control GCaMP signals that were offset from bout initiation by +10 seconds.

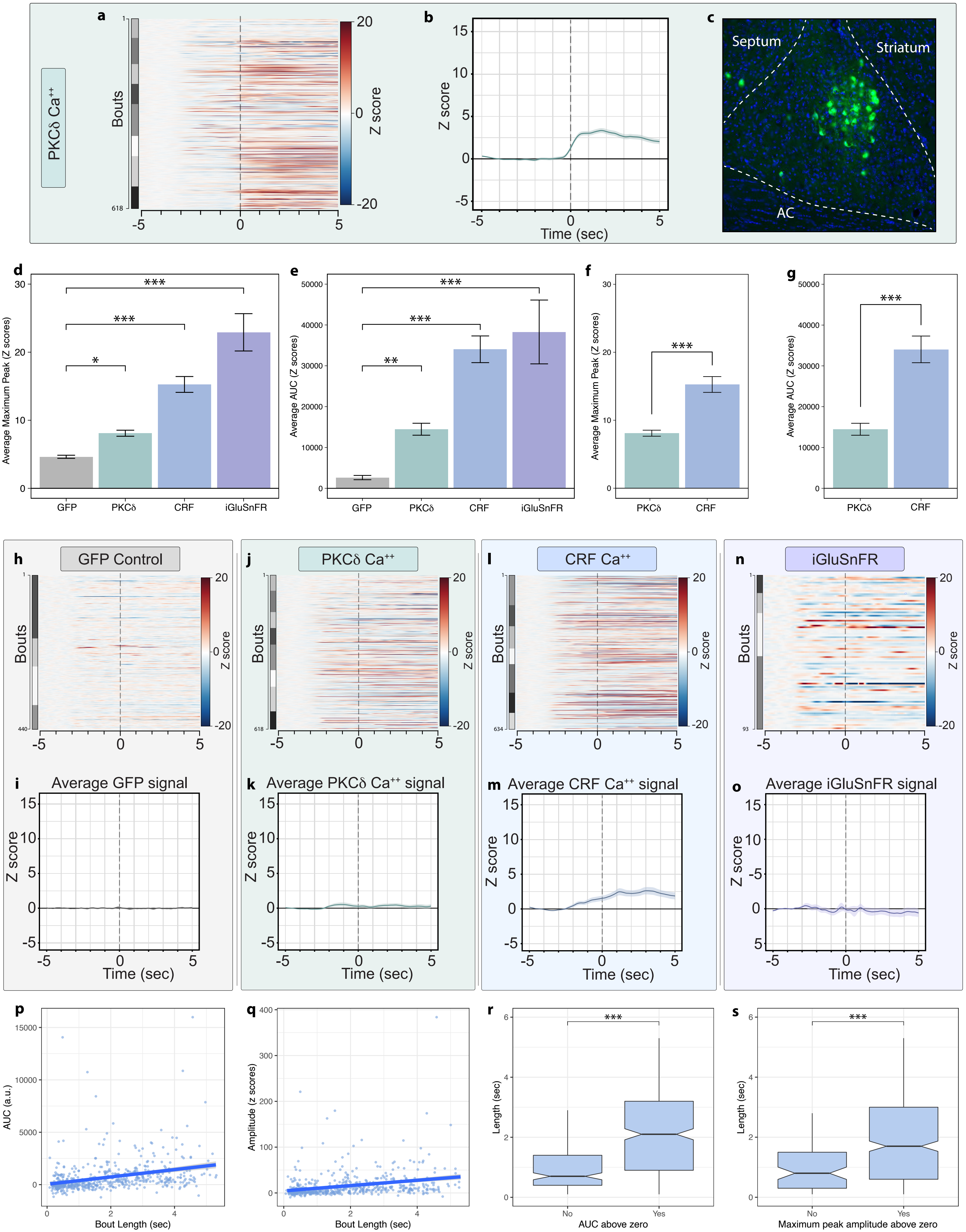

PKCδ Ca++
Bouts
Z score
Time (sec)
Septum
Striatum
AC
Average Maximum Peak (Z scores)
Average AUC (Z scores)
GFP
PKCδ
CRF
iGluSnFR
GFP Control
PKCδ Ca++
CRF Ca++
iGluSnFR
Average GFP signal
Average PKCδ Ca++ signal
Average CRF Ca++ signal
Average iGluSnFR signal
AUC (a.u.)
Bout Length (sec)
Amplitude (z scores)
Length (sec)
No
Yes
AUC above zero
Maximum peak amplitude above zero

Supplementary Figure 2: Fiber photometry controls for Cre-dependent sensors and SF-iGluSnFR.

a-c, Photometry recording from PKCδ-Cre mice that received an injection of Cre-dependent GCaMP in the BNST. **a**, Heatmap of Cre-dependent GCaMP signal in PKCδ-Cre mice that was time-locked bouts. **b**, Average Cre-dependent GCaMP signal PKCδ -Cre mice. Lighter shading: s.e.m. across all bouts. **c**, Example image of Cre-dependent GCaMP expression in PKCδ-Cre mice. **d**, Average maximum peak Z score of PKCδ GCaMP, CRF GCaMP, and SF-iGluSnFR were higher than eGFP controls. One-way ANOVA $F_{(3,1744)} = 40.23$, $P < .0001$; Bonferroni post hoc analysis: *P = 0.034 for eGFP vs. PKCδ -GCaMP; ***P < .0001 for eGFP vs. CRF-GCaMP; ***P < .0001 for eGFP vs. SF-iGluSnFR. **e**, Average AUC Z score of PKCδ GCaMP, CRF GCaMP, and SF-iGluSnFR were higher than eGFP controls. One-way ANOVA $F_{(3,1744)} = 31.98$, $P < .0001$; Bonferroni post hoc analysis: **P = 0. 0056 for eGFP vs. PKCδ-GCaMP; ***P < .0001 for eGFP vs. CRF-GCaMP; ***P < .0001 for eGFP vs. SF-iGluSnFR. **f**, Average maximum peak Z score of CRF-GCaMP signal was higher than PKCδ-GCaMP signal. Welch two-sample t-test, $t_{(811.98)} = 5.7621$, ***P < .0001. **g**, Average AUC Z score of CRF-GCaMP signal was higher than PKCδ-GCaMP signal. Welch two-sample t-test, $t_{(874.12)} = 5.4682$, ***P < .0001. (note: * = p< 0.05, ** = p< 0.01, *** = p< 0.001). **d-g,** PKCδ GCaMP: n = 618 from eight mice, CRF GCaMP: n = 634 from nine mice, SF-iGluSnFR: n = 93 from four mice, and eGFP: n = 440 from four mice. Error bars: s.e.m. across bouts. **h-o,** For all traces, instead of signals being time-locked to the beginning of each bout, they were offset from the bout onset by +10 seconds. **h**, Time-shifted control heatmap for GFP signals in the BNST. **i**, Average ± s.e.m of the time-shifted GFP signals that were offset from bout initiation by +10 seconds. **j**, Time-shifted heatmap for signals from GCaMP expressed in PKCδ cells in the BNST. **k**, Average ± s.e.m of

the offset GCaMP-PKCδ signals. **l**, Time-shifted heatmap for signals from GCaMP expressed in CRF cells in the BNST. **m**, Average ± s.e.m of the offset CRF-GCaMP signals. Slight increase in CRF-GCaMP signal likely reflects CRF cell activity occurring outside of bouts. **n**, Time-shifted heatmap for signals from SF-iGluSnFR expressed in the BNST. **o**, Average ± s.e.m of the offset SF-iGluSnFR signals. **p-s,** Analysis of CRF-GCaMP during the window from 0 to 5 seconds following bout onset. PKCδ GCaMP: n = 618 from eight mice, CRF GCaMP: n = 634 from nine mice, SF-iGluSnFR: n = 93 from four mice, and eGFP: n = 440 from four mice. Median, quartiles, and 1.5 * the interquartile range are indicated with the associated box plot. **p,** AUC vs. bout length (sec). Blue line: the slope of AUC vs. bout length with 95% confidence interval. Pearson correlation: $r_{(571)} = 0.311$, $P < 0.0001$. **q,** Bout length of bouts with an AUC above zero (‘yes’= above zero) vs. those with an AUC not above zero (‘no’= not above zero). Welch two-sample t-test, $t_{(572)} = -10.324$, $P < 0.0001$. **r**, Maximum peak z-score amplitude vs. bout length (sec). Blue line slope of maximum peak vs. bout length with 95% confidence interval. Pearson correlation: $r_{(571)} = 0.2784$, $P < 0.0001$. **s,** Bout length of bouts with a maximum peak above zero vs. those with a maximum peak not above zero. Welch two-sample t-test, Welch two-sample t-test, $t_{(574.64)} = -11.001$, $P < 0.0001$.

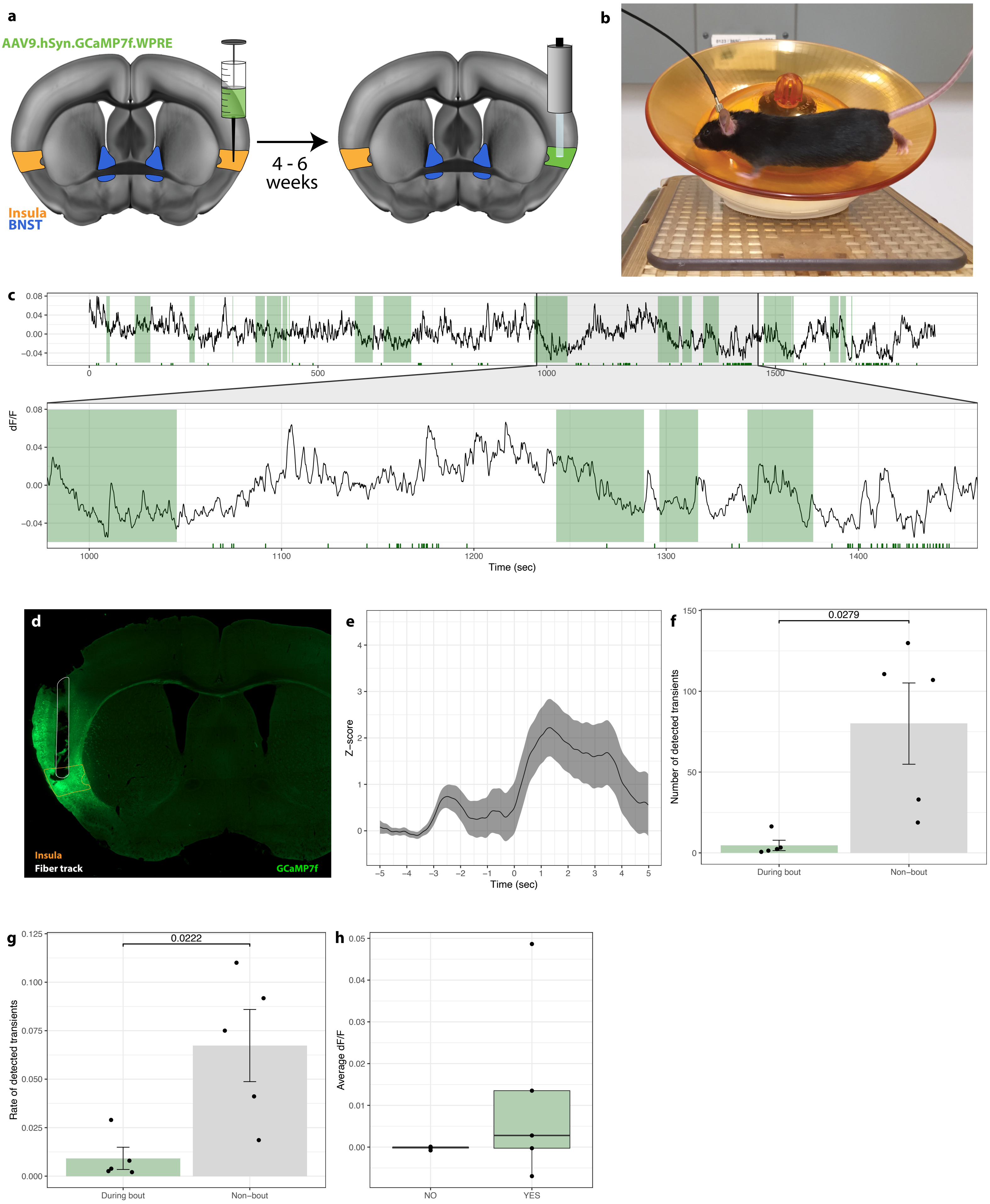
a
AAV9.hSyn.GCaMP7f.WPRE
4 - 6
weeks
Insula
BNST
b
c
0.08
0.04
0.00
−0.04
0
500
1000
1500
dF/F
1000
1100
1200
1300
1400
Time (sec)
d
Insula
Fiber track
GCaMP7f
e
Z−score
Time (sec)
f
0.0279
Number of detected transients
During bout
Non−bout
g
0.0222
Rate of detected transients
During bout
Non−bout
h
Average dF/F
NO
YES

Supplementary Figure 3: Running motion does not increase insular GCaMP signal.

a, Diagram of AAV9.jGCaMP7f injected into the insula and fiberoptic implant into the insular cortex. **b,** Image of a mouse on a saucer-style running wheel with fiber photometry implant and cable. **c,** Representative fiber photometry trace (dF/F) while mouse had access to a running wheel. Top trace: whole 30 minutes. Bottom trace: expansion of the highlighted section of the top trace. Running status is indicated by background shading. Light green: running on wheel. White: not running on wheel. Detected transients are indicated with dark green tick marks at the bottom of the graph. **d,** Representative histological coronal slice. Fluorescent channel: GCaMP7f (green). Outlines: Insula (orange), Fiber track (white). **e,** Average GCaMP signal in mouse insula. Lighter shading: s.e.m. across all bouts. **f,** Aggregate number of detected transients during running vs. non-running portions of the trial. Error bars: s.e.m. across bouts. **g,** Rate of detected transients (Hz) during running vs. non-running portions of the trial. Error bars: s.e.m. across bouts. **h**, Average dF/F signal during running vs. non-running. Median, quartiles, and 1.5 * the interquartile range are indicated with the associated box plot. **f-h**, Each point is the average for one animal.

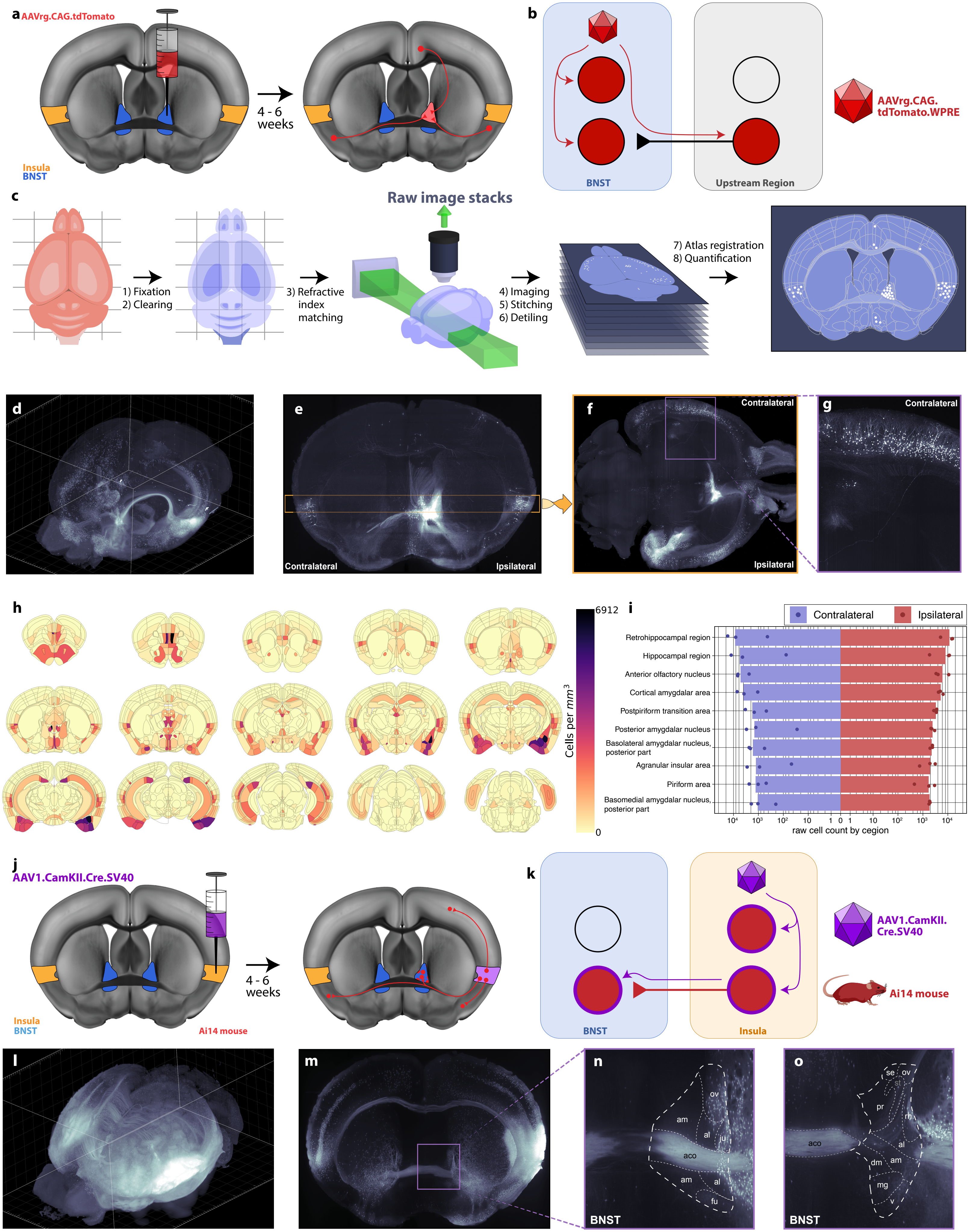

a AAVrg.CAG.tdTomato
4 - 6 weeks
Insula
BNST
b
BNST
Upstream Region
AAVrg.CAG. tdTomato.WPRE
c
Raw image stacks
1) Fixation
2) Clearing
3) Refractive index matching
4) Imaging
5) Stitching
6) Detiling
7) Atlas registration
8) Quantification
d
e
Contralateral
Ipsilateral
f
Contralateral
Ipsilateral
g
Contralateral
h
6912
Cells per mm³
0
i
Contralateral
Ipsilateral
Retrohippocampal region
Hippocampal region
Anterior olfactory nucleus
Cortical amygdalar area
Postpiriform transition area
Posterior amygdalar nucleus
Basolateral amygdalar nucleus, posterior part
Agranular insular area
Piriform area
Basomedial amygdalar nucleus, posterior part
raw cell count by cegion
j
AAV1.CamKII.Cre.SV40
4 - 6 weeks
Insula
BNST
Ai14 mouse
k
BNST
Insula
AAV1.CamKII. Cre.SV40
Ai14 mouse
l
m
n
ov
am
al
ju
aco
am
al
fu
BNST
o
se
ov
st
pr
rh
aco
al
dm
am
mg
v
BNST

Supplementary Figure 4: Inputs to the BNST originate from many stress-implicated regions across the brain, including broad insular inputs that appear to preferentially innervate the dorsal lateral BNST.

a, Diagram of AAVrg coding for tdTomato injection into the BNST. Infected neuron cell bodies, axons, and dendrites are filled with tdTomato. **b**, Schematic of local and upstream viral infection. Cells that do not send projections to the injection site should not be infected. **c**, Overview of light sheet pipeline. **d**, Whole mouse brain after AAVrg-tdTomato injection into the BNST and subsequent light sheet imaging. Olfactory bulbs are in the bottom left corner, and the cerebellum is in the top right corner. **e**, Resampled image in the coronal plane, derived from 3D data set, at the level of the injection site in the BNST. Yellow outline illustrates the transverse plane sliced through for the next image. **f**, Transverse plane through the brain at the level of the insula. **g**, Magnified image of cells in the contralateral insula. Note the readily distinguishable cell bodies to be used for automated cell detection. **h**, Representation of cell density (mm^3) on a composite whole-brain heatmap (averaged from three mice). Cells were semiautonomously quantified using NUGGT[51]. **i**, Top ten labeled upstream regions (based on density of labeled cells) from unilateral AAVrg.tdTomato injection into the BNST in each of 3 mice. Bars: Mean from all three counts per region. Points: Counts from individual animals. Ipsilateral counts on the right in red. Contralateral counts are on the left in blue. The x-axis is an inverse hyperbolic sine transformation. **j**, Diagram of AAV1.Cre anterograde tracing with the injection in the insula of Ai14 mice (n=3). **k**, Overview of AAV1.Cre viral approach in the insula of Ai14 mice. **l**, 3D projection image of whole brain derived from an Ai14 mouse injected in the insula with AAV1.Cre. **m**, Resampled slice in coronal plane at the level of the BNST. tdTomato positive cells are visible bilaterally in both BNSTs. **n**, Magnified view of tdTomato positive cells in the

anterior region of the BNST. Note: For n-o, the morphological borders of subnuclei were charted from the Allen Brain Atlas. **o**, tdTomato positive cells in the posterior region of the BNST.

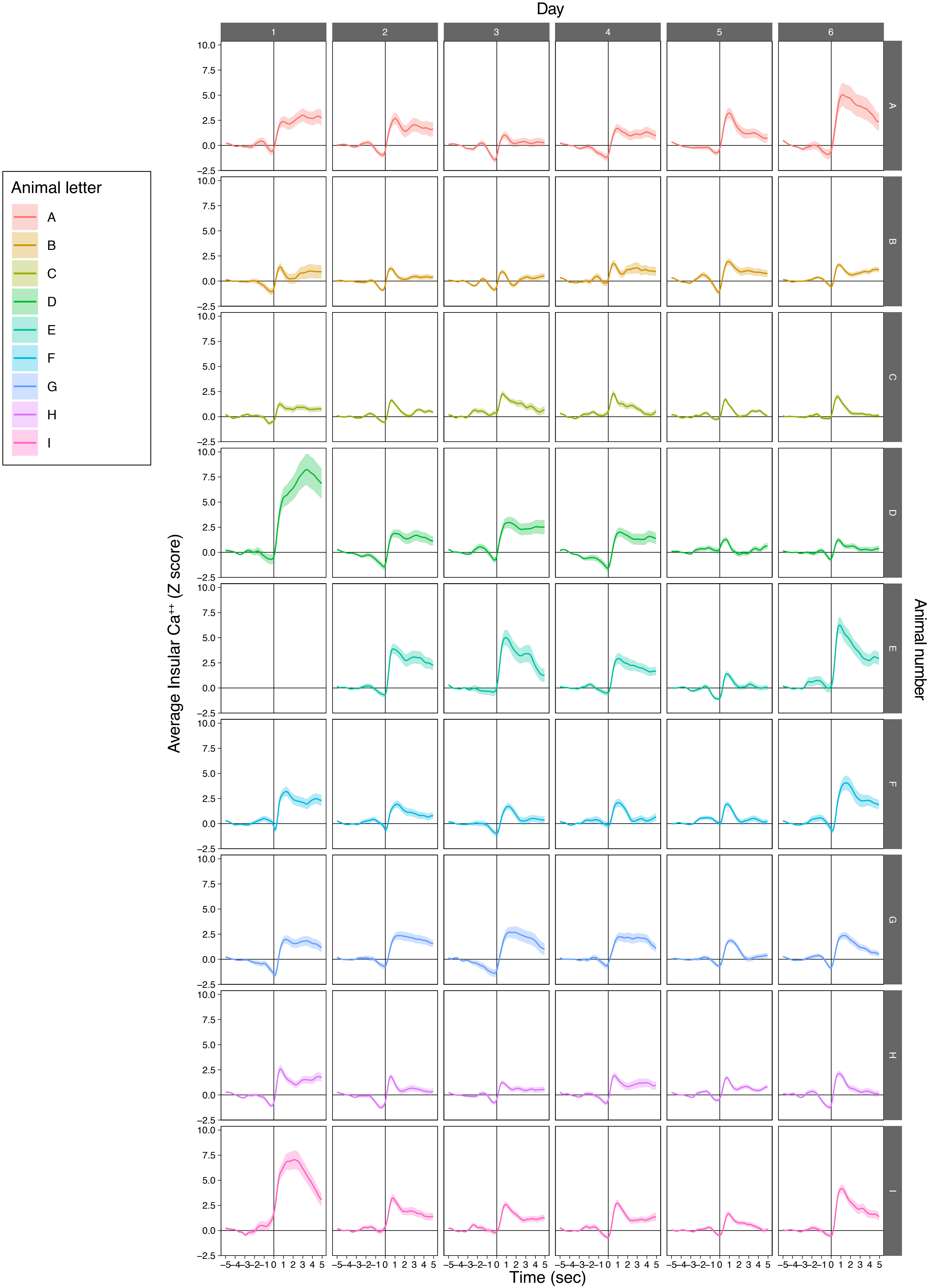

Day
1
2
3
4
5
6
A
B
C
D
E
F
G
H
I
Animal letter
Average Insular Ca⁺⁺ (Z score)
Animal number
Time (sec)
10.0
7.5
5.0
2.5
0.0
−2.5
−5 −4 −3 −2 −1 0 1 2 3 4 5

Supplementary Figure 5: Average time-locked insular GCaMP signal separated by day and animal.

Average signal from individual animals separated by day of experiment. Days 1-6 are separated horizontally. Animals A-I are colored by subject number and are separated vertically. The x-axis is time from -5 seconds preceding to 5 seconds after bout onset. The y-axis is the average GCaMP signal Z score across all bouts for each animal on the indicated day. Lighter shading: s.e.m. across all bouts for that session.

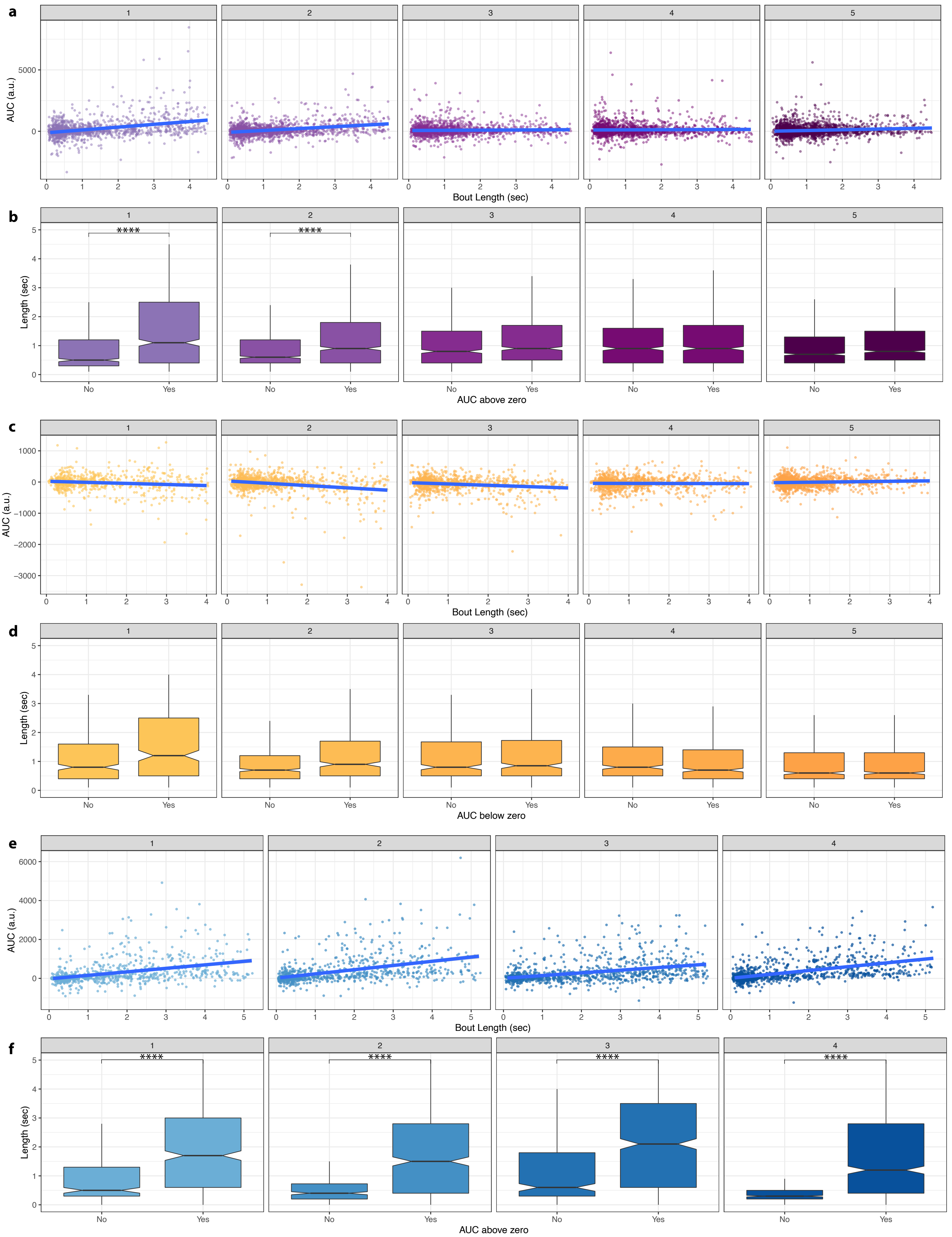
a
b
c
d
e
f
AUC (a.u.)
Bout Length (sec)
Length (sec)
AUC above zero
AUC below zero
No
Yes
****

Supplementary Figure 6: Analysis of AUC vs. bout length for insular GCaMP7f, iGABASnFR, and SF-iGluSnFR after bout onset.

Colors: GCaMP (purple), iGABASnFR (yellow), SF-iGluSnFR (blue). Dots on scatterplots represent the bout length vs. the AUC of the photometry signal for individual bouts on the given day. Blue line: slope of AUC vs. bout length with 95% confidence interval. Note: correlation coefficients are indicated in Fig. 3g. Boxplots compare bouts with an AUC above zero vs. those with an AUC not above zero. Notch indicates confidence interval around the median. ****$P < 0.0001$. **a,** GCaMP: AUC vs. bout length (sec) from days one to five. Rimonabant given on day five. **b,** GCaMP: Bout length of bouts with an AUC above zero (‘yes’= above zero) vs. those with an AUC not above zero (‘no’= not above zero). Rimonabant given on day five. Two-way ANOVA, main effect of above vs. below zero $F_{(1, 11126)} = 114.36$, $P < 0.0001$; main effect of date $F_{(1, 11126)} = 33.57$, $P < 0.0001$; Bonferroni post hoc analysis for above vs. below zero denoted on graph. Number of bouts equal to the points in corresponding plot from S6a from a total of nine animals. Median, quartiles, and 1.5 * the interquartile range are indicated with the associated box plot. **c,** iGABASnFR: AUC vs. bout length (sec) from days one to five. Rimonabant given on day five. Blue line slope of AUC vs. bout length with 95% confidence interval. **d,** iGABASnFR: Bout length of bouts with an AUC below zero (‘yes’= below zero) vs. those with an AUC not below zero (‘no’= not below zero). Rimonabant given on day five. Two-way ANOVA, main effect of above vs. below zero $F_{(1, 5145)} = 0.518$, $P = 0.472$; main effect of date $F_{(1, 5145)} = 62.144$, $P < 0.0001$; Bonferroni post hoc analysis for above vs. below zero denoted on graph. Number of bouts equal to the points in corresponding plot from S6c from a total of five animals. Median, quartiles, and 1.5 * the interquartile range are indicated with the associated box plot. **e,** SF-iGluSnFR: AUC vs. bout length (sec) from days one to four. Blue line slope of AUC vs. bout

length with 95% confidence interval. **f,** SF-iGluSnFR: Bout length of bouts with an AUC above zero (‘yes’= above zero) vs. those with an AUC not above zero (‘no’= not above zero). Two-way ANOVA, main effect of above vs. below zero $F_{(1, 3641)} = 15.233$, $P < 0.0001$; main effect of date $F_{(1, 3641)} = 7.422$, $P = 0.00647$; Bonferroni post hoc analysis for above vs. below zero denoted on graph. Number of bouts equal to the points in corresponding plot from S6e from a total of ten animals. Median, quartiles, and 1.5 * the interquartile range are indicated with the associated box plot.

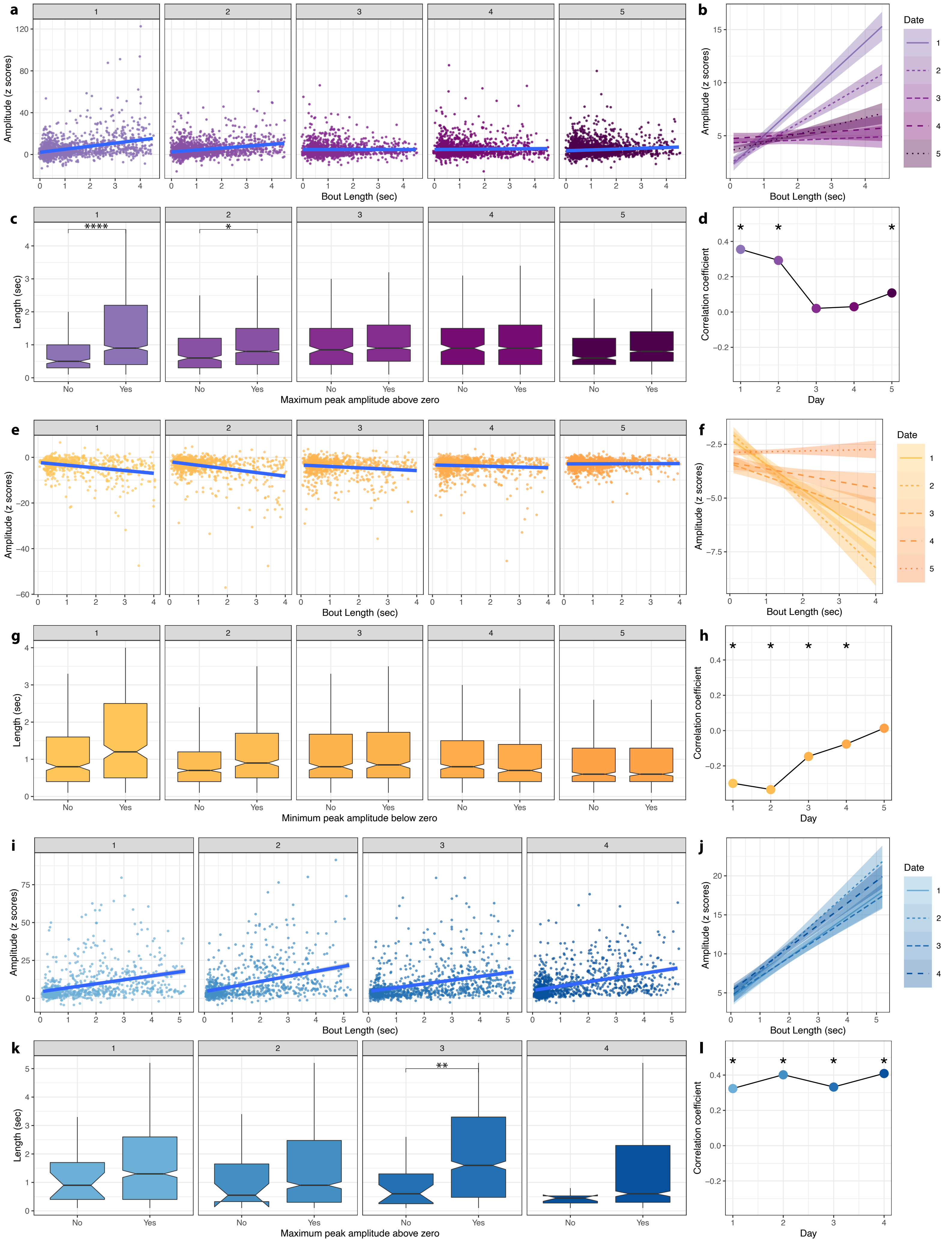

a
b
c
d
e
f
g
h
i
j
k
l
Amplitude (z scores)
Bout Length (sec)
Date
Length (sec)
Maximum peak amplitude above zero
Correlation coefficient
Day
Minimum peak amplitude below zero
No
Yes
****
**

Supplementary Figure 7: Analysis of maximum peak photometry amplitude vs. bout length for insular GCaMP7f, iGABASnFR, and SF-iGluSnFR following bout onset.

Colors: **a-d,** GCaMP (purple). **e-h,** iGABASnFR (yellow). **i-l,** SF-iGluSnFR (blue). Dots on scatterplots represent the bout length vs. the photometry signal's peak amplitude for individual bouts on the given day. Blue line: slope of peak amplitude vs. bout length with 95% confidence interval. Slopes for each sensor are overlayed for comparison across days. Boxplots compare bouts with a peak amplitude above zero vs. those with a peak amplitude not above zero. Notch indicates confidence interval around the median. Correlation coefficients are shown across days. *P < 0.05, **P < 0.01, ****P < 0.0001. **a,** GCaMP: maximum peak amplitude vs. bout length (sec) from days one to five. Rimonabant given on day five. **b,** GCaMP: overlayed slopes of maximum peak amplitude vs. bout across days length from Fig. S7a. **c,** GCaMP: Bout length of bouts with a maximum peak amplitude above zero (‘yes’= above zero) vs. those with a maximum peak amplitude not above zero (‘no’= not above zero). Rimonabant given on day five. Two-way ANOVA, main effect of above vs. below zero $F_{(1, 11126)} = 46.29$, $P < 0.0001$; main effect of date $F_{(1, 11126)} = 34.61$, $P < 0.0001$; Bonferroni post hoc analysis for above vs. below zero denoted on graph. Number of bouts equal to the points in corresponding plot from S7a from a total of nine animals. Median, quartiles, and 1.5 * the interquartile range are indicated with the associated box plot. **d,** GCaMP: correlation coefficients for maximum peak amplitude vs. bout length across days from Fig. S7a. **e,** iGABASnFR: minimum peak amplitude vs. bout length (sec) from days one to five. Rimonabant given on day five. **f,** iGABASnFR: overlayed slopes of minimum peak amplitude vs. bout across days length from Fig. S7e. **g,** iGABASnFR: Bout length of bouts with a minimum peak amplitude above zero (‘yes’= below zero) vs. those with n maximum peak amplitude not above zero (‘no’= not below zero). Two-way ANOVA, main

effect of above vs. below zero $F_{(1, 5145)} = 1.296$, $P = 0.255$; main effect of date $F_{(1, 5145)} = 61.397$, $P < 0.0001$; Bonferroni post hoc analysis for above vs. below zero denoted on graph. Number of bouts equal to the points in corresponding plot from S7e from a total of five animals. Median, quartiles, and 1.5 * the interquartile range are indicated with the associated box plot. **h,** iGABASnFR: correlation coefficients for minimum peak amplitude vs. bout length across days from Fig. S7e. **i,** SF-iGluSnFR: maximum peak amplitude vs. bout length (sec) from days one to five. Rimonabant given on day five. **j,** SF-iGluSnFR: overlayed slopes of maximum peak amplitude vs. bout across days length from Fig. S7i. **k,** SF-iGluSnFR: bout length of bouts with maximum peak amplitude above zero (‘yes’= above zero) vs. those with a maximum peak amplitude not above zero (‘no’= not above zero). Number of bouts equal to the points in corresponding plot from S7i from a total of ten animals. Two-way ANOVA, main effect of above vs. below zero $F_{(1, 3641)} = 442.62$, $P < 0.0001$; main effect of date $F_{(1, 3641)} = 11.13$, $P = 0.000856$; Bonferroni post hoc analysis for above vs. below zero denoted on graph. Median, quartiles, and 1.5 * the interquartile range are indicated with the associated box plot. **l,** SF-iGluSnFR: correlation coefficients for maximum peak amplitude vs. bout length across days from Fig. S7i.

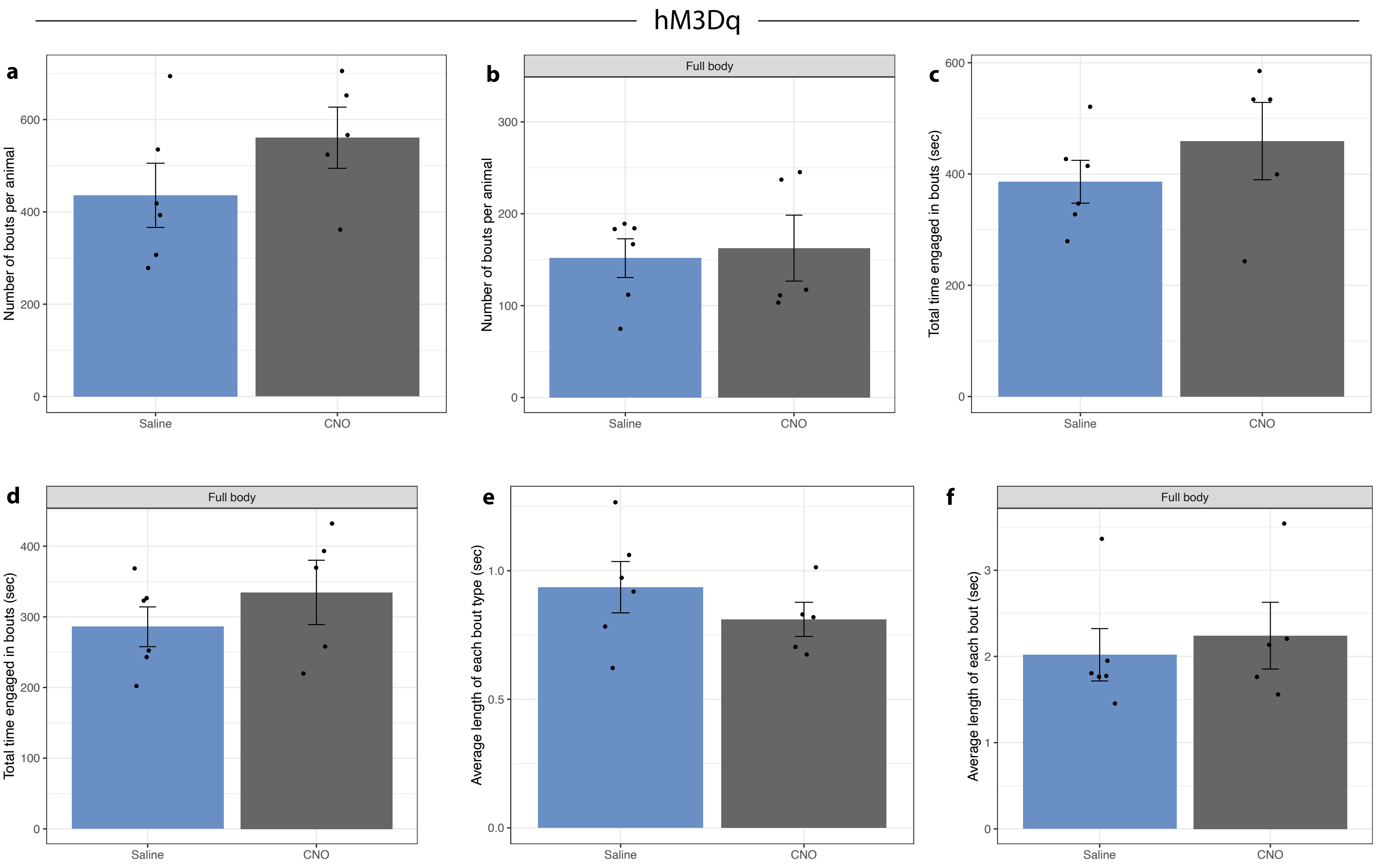

hM3Dq
a
Number of bouts per animal
0
200
400
600
Saline
CNO
b
Full body
Number of bouts per animal
0
100
200
300
Saline
CNO
c
Total time engaged in bouts (sec)
0
200
400
600
Saline
CNO
d
Full body
Total time engaged in bouts (sec)
0
100
200
300
400
Saline
CNO
e
Average length of each bout type (sec)
0.0
0.5
1.0
Saline
CNO
f
Full body
Average length of each bout (sec)
0
1
2
3
Saline
CNO

Supplementary Figure 8: Restraint bout data when chemogenetically activating insula$^{\rightarrow BNST}$ cells.

a, Average number of bouts (all types) during the 30-minute restraint with CNO (grey) and saline (blue) treated animals. Welch two-sample t-test, $t_{(8.98)}$ = -1.4359, P = 0.1849. **b,** Average number of full body bouts during the 30-minute restraint of CNO (grey) and saline (blue) treated animals. Welch two-sample t-test, $t_{(6.70)}$ = -0.292, P = 0.779. **c**, Average time engaged in bouts (all types) during the 30-minute restraint of CNO (grey) and saline (blue) treated animals. Welch two-sample t-test, $t_{(6.45)}$ = -1.024, P = 0.3428. **d,** Average time engaged in full body bouts during the 30-minute restraint of CNO (grey) and saline (blue) treated animals. Welch two-sample t-test, $t_{(6.94)}$ = -1.0097, P = 0.3465. **c**, Average time engaged in bouts (all types) during the 30-minute restraint of CNO (grey) and saline (blue) treated animals. Welch two-sample t-test, $t_{(6.45)}$ = -1.024, P = 0.3428. **d,** Average number of full body bouts during the 30-minute restraint of CNO (grey) and saline (blue) treated animals. Welch two-sample t-test, $t_{(6.94)}$ = -1.0097, P = 0.3465. **e**, Average time length of each bout (all types) during the 30-minute restraint of CNO (grey) and saline (blue) treated animals. Welch two-sample t-test, $t_{(8.29)}$ = 1.1504, P = 0.2821. **f,** Average number of full body bouts during the 30-minute restraint of CNO (grey) and saline (blue) treated animals. Welch two-sample t-test, $t_{(8.11)}$ = -0.50056, P = 0.63. **a-f**, CNO (n = 5 animals), saline (n = 6 animals). Error bars: s.e.m. across bouts.

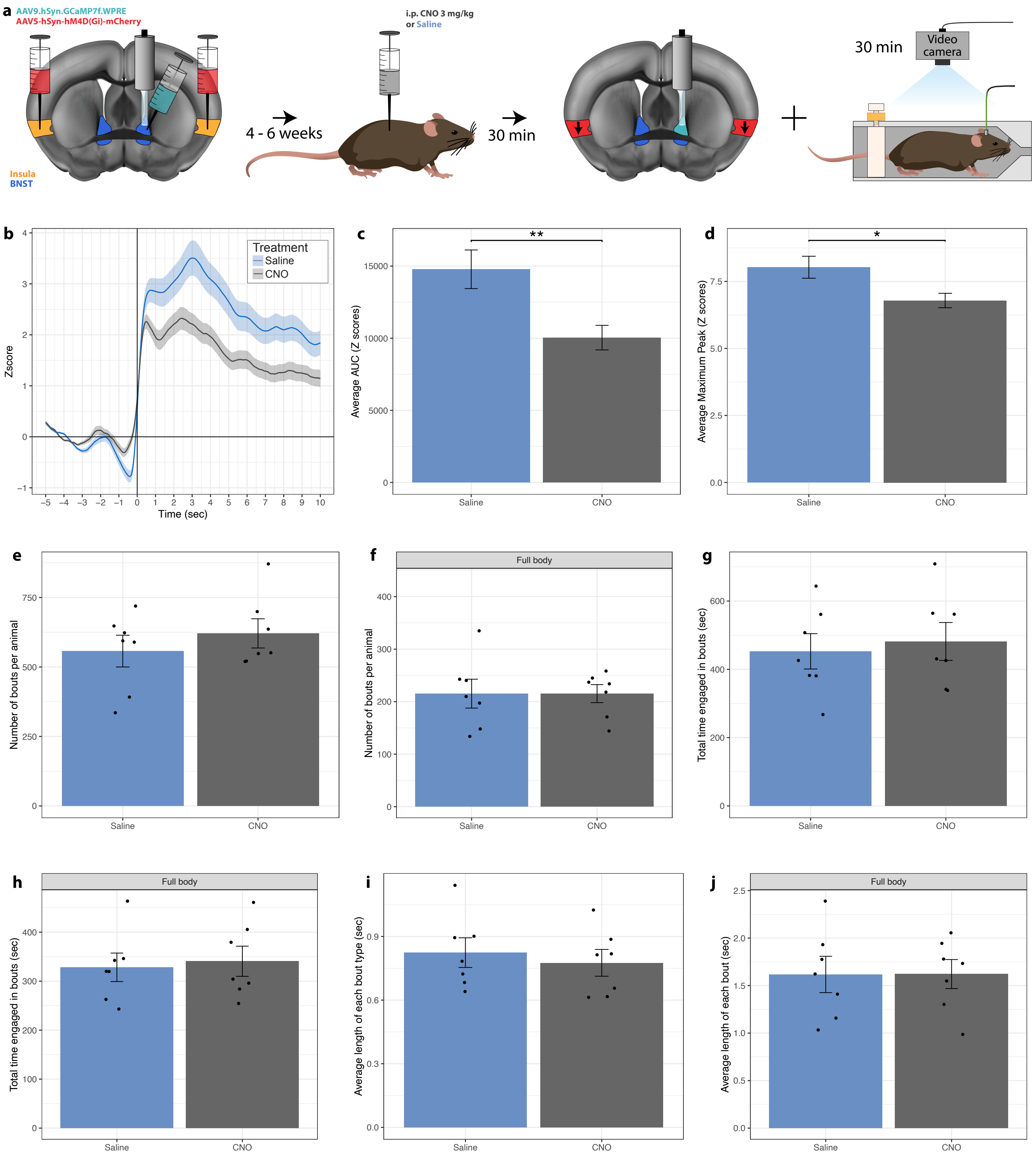
a
AAV9.hSyn.GCaMP7f.WPRE
AAV5-hSyn-hM4D(Gi)-mCherry
Insula
BNST
4 - 6 weeks
i.p. CNO 3 mg/kg
or Saline
30 min
30 min
Video camera
b
Treatment
Saline
CNO
Zscore
Time (sec)
c
**
Average AUC (Z scores)
Saline
CNO
d
*
Average Maximum Peak (Z scores)
Saline
CNO
e
Number of bouts per animal
Saline
CNO
f
Full body
Number of bouts per animal
Saline
CNO
g
Total time engaged in bouts (sec)
Saline
CNO
h
Full body
Total time engaged in bouts (sec)
Saline
CNO
i
Average length of each bout type (sec)
Saline
CNO
j
Full body
Average length of each bout (sec)
Saline
CNO

Supplementary Figure 9: Insular G_i-DREADD activation decreases BNST transient size but does not change struggling bouts.

a, Diagram of experimental timeline. Mice received bilateral insula injections with a G_i-DREADD (hM4Di) coding virus and unilateral BNST injections coding for GCaMP7f. A fiber photometry implant was also placed in the same BNST. Four to six weeks after fiber implantation, animals were injected with CNO (i.p. 3 mg/kg). Thirty minutes later, animals were placed in a restraint stress device and recorded for automated behavioral analysis. **b**, Average of all full body bout-associated signals from GCaMP signal in CNO (grey) and saline (blue) treated animals. Lighter shading: s.e.m. across bouts. **c,** Average area under the curve (AUC) of Z scores from all bouts of CNO (grey) and saline (blue) treated animals. Welch two-sample t-test, $t_{(2220.6)} = 2.9892$, **P = 0.0028. Error bars: s.e.m. across bouts. **d,** Average Maximum Peak Z score from all bouts of CNO (grey) and saline (blue) treated animals. Welch two-sample t-test, $t_{(2265.9)} = 2.5376$, *P = 0.0112. Error bars: s.e.m. across bouts. **e**, Average number of bouts (all types) during the 30-minute restraint of CNO (grey) and saline (blue) treated animals. Welch two-sample t-test, $t_{(11.913)} = -0.88636$, P = 0.393. **c-d, (**CNO: n = 1492 bouts from seven animals, saline: n = 1285 bouts from seven animals.) **f,** Average number of full body bouts during the 30-minute restraint of CNO (grey) and saline (blue) treated animals. Welch two-sample t-test, $t_{(10.053)} = 0$, P = 1. **g**, Average time engaged in bouts (all types) during the 30-minute restraint of CNO (grey) and saline (blue) treated animals. Welch two-sample t-test, $t_{(11.942)} = -0.41159$, P = 0.6879. **h,** Average time engaged in full body bouts during the 30-minute restraint of CNO (grey) and saline (blue) treated animals. Welch two-sample t-test, $t_{(11.96)} = -0.3151$, P = 0.7581. **i**, Average time length of each bout (all types) during the 30-minute restraint of CNO (grey) and saline (blue) treated animals. Welch two-sample t-test, $t_{(11.874)} = 0.55116$, P = 0.5917. **j,** Average

number of full body bouts during the 30-minute restraint of CNO (grey) and saline (blue) treated animals. Welch two-sample t-test, $t_{(11.446)} = -0.01907$, $P = 0.9851$. **e-j**, Error bars: s.e.m. across animals.

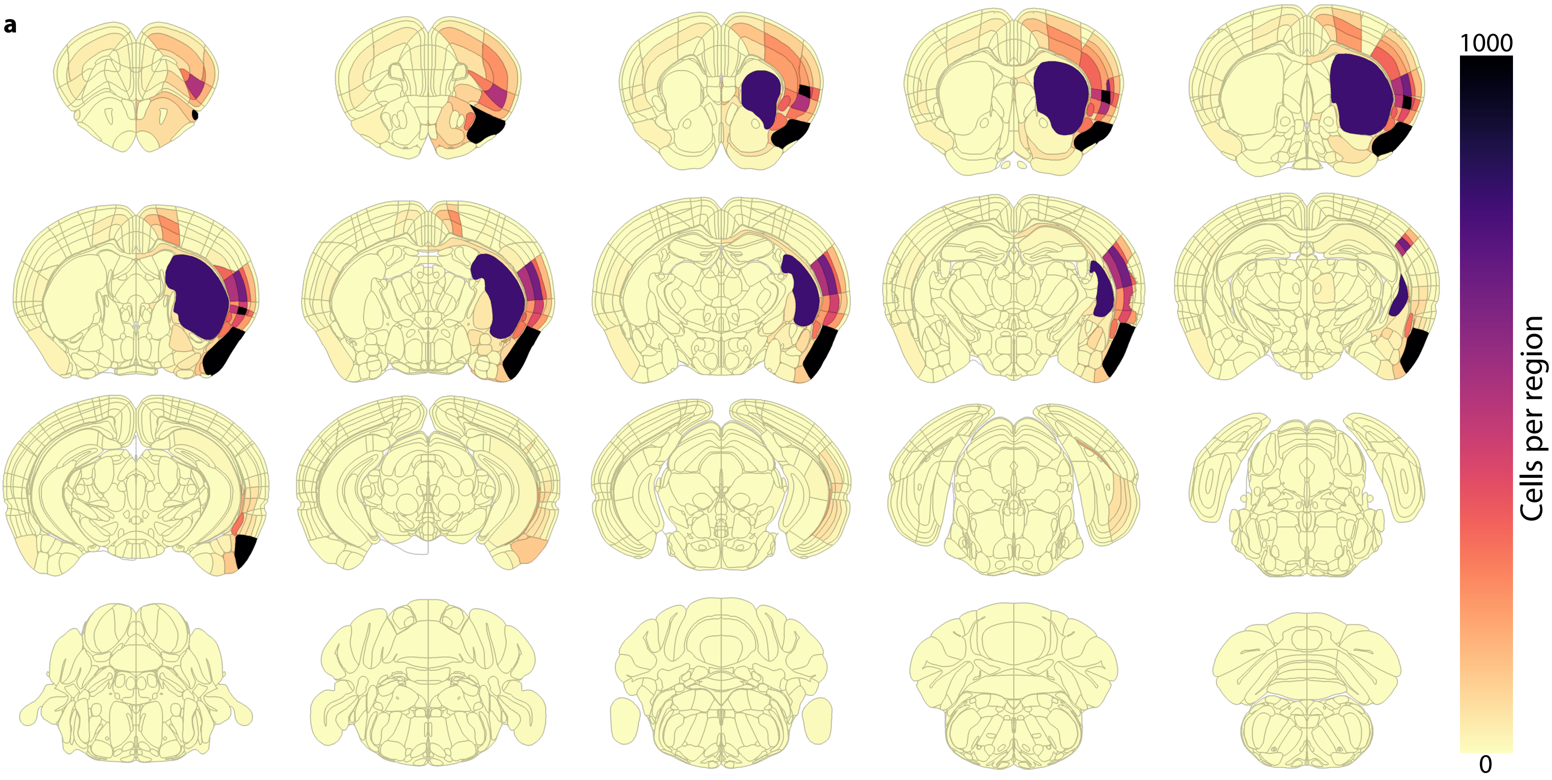

TRIO insula$^{\rightarrow BNST}$ control network

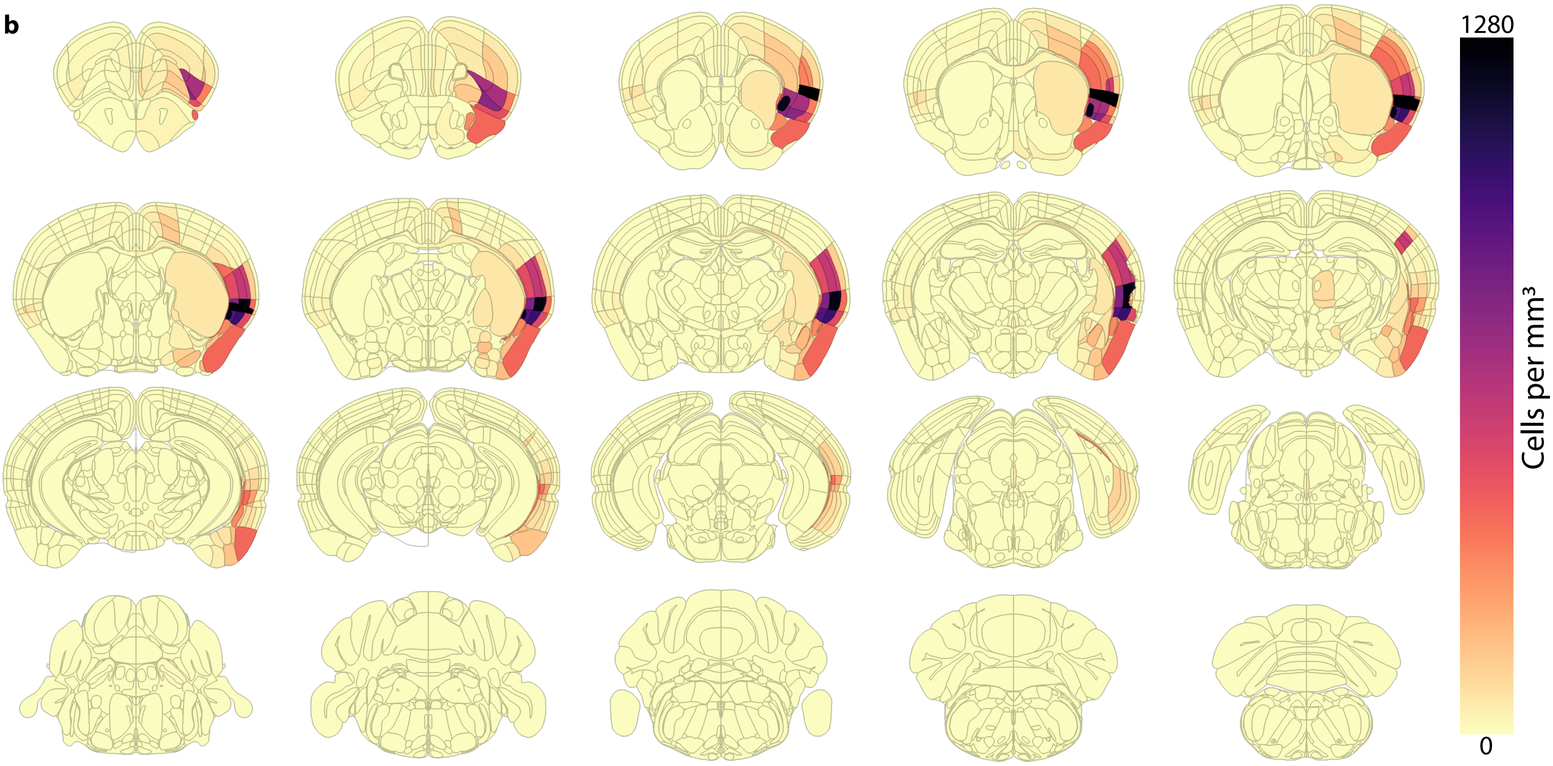

Supplementary Figure 10: Expanded maps of the control network for the insula$^{\rightarrow BNST}$ pathway.

a, Raw cell counts of labeled cells represented as composite whole-brain heatmap showing relative number of cells that project to insula$^{\rightarrow BNST}$ neurons (averaged from five mice). **b**, Complete set of levels from composite whole-brain heatmap showing regional cell density in mm^3 of cell per region that project to insula$^{\rightarrow BNST}$ neurons (averaged from five mice).

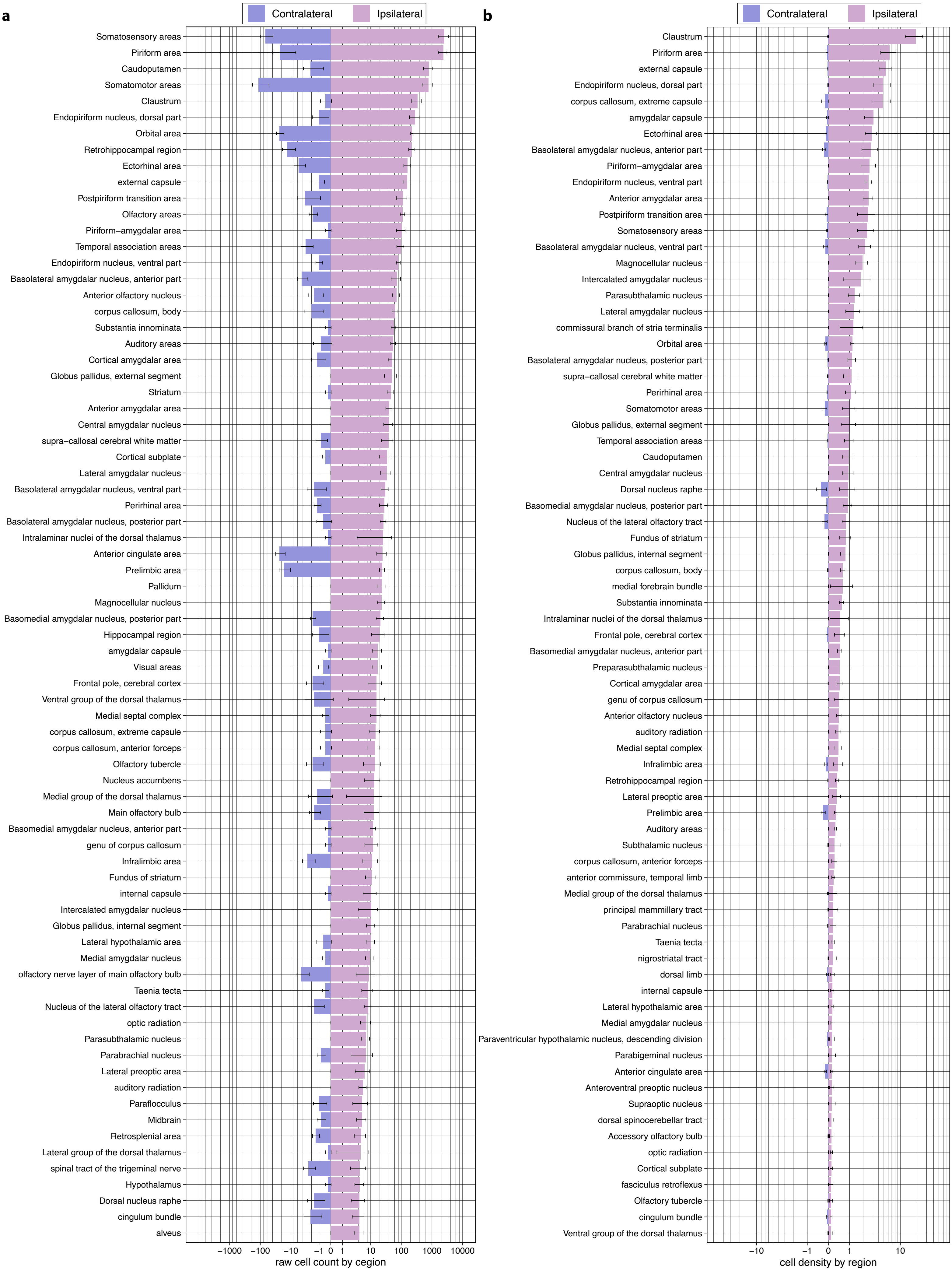
a
Contralateral
Ipsilateral
Somatosensory areas
Piriform area
Caudoputamen
Somatomotor areas
Claustrum
Endopiriform nucleus, dorsal part
Orbital area
Retrohippocampal region
Ectorhinal area
external capsule
Postpiriform transition area
Olfactory areas
Piriform−amygdalar area
Temporal association areas
Endopiriform nucleus, ventral part
Basolateral amygdalar nucleus, anterior part
Anterior olfactory nucleus
corpus callosum, body
Substantia innominata
Auditory areas
Cortical amygdalar area
Globus pallidus, external segment
Striatum
Anterior amygdalar area
Central amygdalar nucleus
supra−callosal cerebral white matter
Cortical subplate
Lateral amygdalar nucleus
Basolateral amygdalar nucleus, ventral part
Perirhinal area
Basolateral amygdalar nucleus, posterior part
Intralaminar nuclei of the dorsal thalamus
Anterior cingulate area
Prelimbic area
Pallidum
Magnocellular nucleus
Basomedial amygdalar nucleus, posterior part
Hippocampal region
amygdalar capsule
Visual areas
Frontal pole, cerebral cortex
Ventral group of the dorsal thalamus
Medial septal complex
corpus callosum, extreme capsule
corpus callosum, anterior forceps
Olfactory tubercle
Nucleus accumbens
Medial group of the dorsal thalamus
Main olfactory bulb
Basomedial amygdalar nucleus, anterior part
genu of corpus callosum
Infralimbic area
Fundus of striatum
internal capsule
Intercalated amygdalar nucleus
Globus pallidus, internal segment
Lateral hypothalamic area
Medial amygdalar nucleus
olfactory nerve layer of main olfactory bulb
Taenia tecta
Nucleus of the lateral olfactory tract
optic radiation
Parasubthalamic nucleus
Parabrachial nucleus
Lateral preoptic area
auditory radiation
Paraflocculus
Midbrain
Retrosplenial area
Lateral group of the dorsal thalamus
spinal tract of the trigeminal nerve
Hypothalamus
Dorsal nucleus raphe
cingulum bundle
alveus
−1000 −100 −10 −1 0 1 10 100 1000 10000
raw cell count by cegion
b
Contralateral
Ipsilateral
Claustrum
Piriform area
external capsule
Endopiriform nucleus, dorsal part
corpus callosum, extreme capsule
amygdalar capsule
Ectorhinal area
Basolateral amygdalar nucleus, anterior part
Piriform−amygdalar area
Endopiriform nucleus, ventral part
Anterior amygdalar area
Postpiriform transition area
Somatosensory areas
Basolateral amygdalar nucleus, ventral part
Magnocellular nucleus
Intercalated amygdalar nucleus
Parasubthalamic nucleus
Lateral amygdalar nucleus
commissural branch of stria terminalis
Orbital area
Basolateral amygdalar nucleus, posterior part
supra−callosal cerebral white matter
Perirhinal area
Somatomotor areas
Globus pallidus, external segment
Temporal association areas
Caudoputamen
Central amygdalar nucleus
Dorsal nucleus raphe
Basomedial amygdalar nucleus, posterior part
Nucleus of the lateral olfactory tract
Fundus of striatum
Globus pallidus, internal segment
corpus callosum, body
medial forebrain bundle
Substantia innominata
Intralaminar nuclei of the dorsal thalamus
Frontal pole, cerebral cortex
Basomedial amygdalar nucleus, anterior part
Preparasubthalamic nucleus
Cortical amygdalar area
genu of corpus callosum
Anterior olfactory nucleus
auditory radiation
Medial septal complex
Infralimbic area
Retrohippocampal region
Lateral preoptic area
Prelimbic area
Auditory areas
Subthalamic nucleus
corpus callosum, anterior forceps
anterior commissure, temporal limb
Medial group of the dorsal thalamus
principal mammillary tract
Parabrachial nucleus
Taenia tecta
nigrostriatal tract
dorsal limb
internal capsule
Lateral hypothalamic area
Medial amygdalar nucleus
Paraventricular hypothalamic nucleus, descending division
Parabigeminal nucleus
Anterior cingulate area
Anteroventral preoptic nucleus
Supraoptic nucleus
dorsal spinocerebellar tract
Accessory olfactory bulb
optic radiation
Cortical subplate
fasciculus retroflexus
Olfactory tubercle
cingulum bundle
Ventral group of the dorsal thalamus
−10 −1 0 1 10
cell density by region

Supplementary Figure 11: Expanded data of the control network for the insula$^{\rightarrow BNST}$ pathway using light sheet imaging.

a, Raw cell counts from top 75 labeled upstream regions from unilateral insula$^{\rightarrow BNST}$ cells in five mice. Bars: Mean from all three counts per region. Error bars: s.e.m. across animals. Ipsilateral counts on the right in purple. Contralateral counts are on the left in blue. The x-axis is an inverse hyperbolic sine transformation. **b**, Density (in mm^3) of top 75 labeled upstream regions from unilateral insula$^{\rightarrow BNST}$ cells in each of five mice.

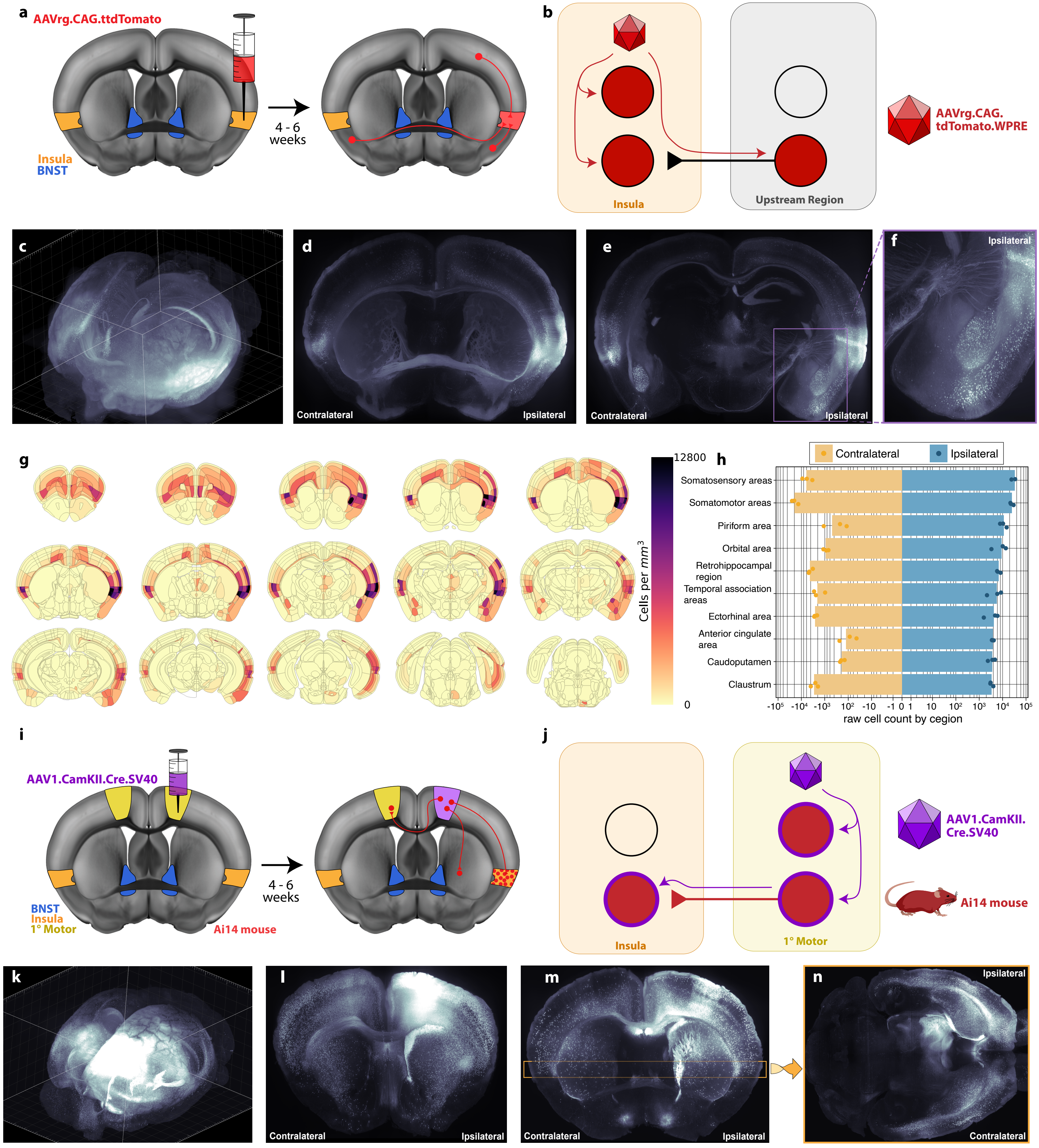

a
AAVrg.CAG.ttdTomato
4 - 6 weeks
Insula
BNST
b
Insula
Upstream Region
AAVrg.CAG. tdTomato.WPRE
c
d
Contralateral
Ipsilateral
e
Contralateral
Ipsilateral
f
Ipsilateral
g
12800
0
Cells per mm^3
h
Contralateral
Ipsilateral
Somatosensory areas
Somatomotor areas
Piriform area
Orbital area
Retrohippocampal region
Temporal association areas
Ectorhinal area
Anterior cingulate area
Caudoputamen
Claustrum
-10^5 -10^4 -10^3 10^2 -10 -1 0 1 10 10^2 10^3 10^4 10^5
raw cell count by cegion
i
AAV1.CamKII.Cre.SV40
4 - 6 weeks
BNST
Insula
1° Motor
Ai14 mouse
j
Insula
1° Motor
AAV1.CamKII. Cre.SV40
Ai14 mouse
k
l
Contralateral
Ipsilateral
m
Contralateral
Ipsilateral
n
Ipsilateral
Contralateral

Supplementary Figure 12: The insular region that projects densely to the BNST receives input from motor cortex.

a, Diagram of AAVrg coding for tdTomato injection into the insula. Transduced neuron cell bodies, axons, and dendrites are labeled with tdTomato. **b**, Schematic of local insular and upstream viral infection. Cells that do not send projections to the injection site should not be labeled. **c**, 3D projection image of cellular labeling in whole mouse brain after AAVrg-tdTomato injection into the insula and subsequent light sheet imaging. Olfactory bulbs are in the bottom left corner, and the cerebellum is in the top right corner. **d**, Resampled image in coronal plane through the anterior BNST. The lack of labeled cells in the BNST indicates minimal direct BNST projections to the insula. **e**, Resampled image in coronal plane through the amygdala. The purple box illustrates the zoomed-in region in the following image. **f**, Magnified image of cells in the ipsilateral amygdala. **g**, Coronal sections from composite whole-brain heatmap (averaged from three mice with AAVrg.tdTomato injected into the insula) of cell density in mm^3. Cells were semiautonomously quantified using NUGGT[51]. **h**, Top ten labeled upstream regions from AAVrg.tdTomato injection into unilateral insula of 3 mice. Bars: Mean from all three counts per region. Points: Counts from individual animals. Ipsilateral counts on the right in teal. Contralateral counts are on the left in goldenrod. The x-axis is an inverse hyperbolic sine transformation. **i**, Diagram of AAV1.Cre injected into the motor cortex of an Ai14 mouse with downstream cells expressing tdTomato. **j**, Schematic of AAV1.Cre labeling downstream neurons. **k**, 3D projection image of a whole AAV1.Cre mouse (Ai14) brain injected in the motor cortex. Olfactory bulbs are in the bottom left corner, and the cerebellum is in the top right corner. **l**, Coronal view showing the injection site in motor cortex. **m**, Coronal view at the level of the BNST. The yellow outline indicates the transverse section through the insula shown in the

following image. **n**, Resampled image in the horizonal plane at the level of the insula (cerebellum = left, olfactory bulbs = right).
